# Informing the Structure of Executive Function in Children: A Meta-Analysis of Functional Neuroimaging Data

**DOI:** 10.3389/fnhum.2017.00154

**Published:** 2017-04-07

**Authors:** Róisín McKenna, T. Rushe, Kate A. Woodcock

**Affiliations:** School of Psychology, Queen's UniversityBelfast, Northern Ireland

**Keywords:** executive function, fMRI, children, ALE meta-analysis, inhibition, switching, updating, cognitive control

## Abstract

The structure of executive function (EF) has been the focus of much debate for decades. What is more, the complexity and diversity provided by the developmental period only adds to this contention. The development of executive function plays an integral part in the expression of children's behavioral, cognitive, social, and emotional capabilities. Understanding how these processes are constructed during development allows for effective measurement of EF in this population. This meta-analysis aims to contribute to a better understanding of the structure of executive function in children. A coordinate-based meta-analysis was conducted (using BrainMap GingerALE 2.3), which incorporated studies administering functional magnetic resonance imaging (fMRI) during inhibition, switching, and working memory updating tasks in typical children (aged 6–18 years). The neural activation common across all executive tasks was compared to that shared by tasks pertaining only to inhibition, switching or updating, which are commonly considered to be fundamental executive processes. Results support the existence of partially separable but partially overlapping inhibition, switching, and updating executive processes at a neural level, in children over 6 years. Further, the shared neural activation across all tasks (associated with a proposed “unitary” component of executive function) overlapped to different degrees with the activation associated with each individual executive process. These findings provide evidence to support the suggestion that one of the most influential structural models of executive functioning in adults can also be applied to children of this age. However, the findings also call for careful consideration and measurement of both specific executive processes, and unitary executive function in this population. Furthermore, a need is highlighted for a new systematic developmental model, which captures the integrative nature of executive function in children.

## Introduction

Executive function (EF) is an umbrella term for a number of inter-related cognitive processes needed for purposeful, goal-orientated behavior (Anderson, 2001; Lerner and Lonigan, 2014). EF enables the regulation and monitoring of high level cognitive resources and is usually employed in novel situations (Shallice, 1988; Stuss, 1992). Cognitive processes associated with EF include planning, problem-solving, novel thinking, and the ability to adapt behavior to the changing environment (Zelazo et al., 2003; Banich, 2004). Additionally, EF performance reliably predicts many intellectual and social competencies, such as school readiness (Welsh et al., 2010), early literacy, and numeracy attainment (Blair and Razza, 2007), later school accomplishment (Checa and Rueda, 2011) and social understanding (Riggs et al., 2006). The terms “executive function” and “cognitive control” are regularly used interchangeably in the literature (MacDonald, 2008; Lenartowicz et al., 2010). However—although our position supports this view—for the purpose of clarity and because our work draws heavily on perspectives that have used the “executive function” term, in this paper this term will be used throughout. Broadly speaking, impairment in EF has been linked to behavioral problems, and is evidenced in individuals with neurodevelopmental disorders including reading disorders, attention deficit hyperactivity disorder (ADHD), autism and several genetic syndromes, including for example, Prader-Willi syndrome (Booth et al., 2003; Kenworthy et al., 2008; Woodcock et al., 2009, 2010; Visser et al., 2015; Danforth et al., 2016). Despite this, findings in relation to how EF may be linked to clinically relevant behavior remain largely inconsistent. The focus of the present meta-analysis is to investigate the neural structure of EF in children during typical development. Such knowledge is necessary to elucidate the executive underpinnings of clinically relevant behavior in individuals with neurodevelopmental disorders.

There has been much debate on how executive function is structured, for example on how far individual executive processes may reflect manifestations of a single EF capacity or of multiple component processes (Miyake et al., 2000; Best et al., 2009). However, a leading theory, known as the integrative model (Miyake et al., 2000), consolidates such unitary and dissociative views. Importantly, the processes considered in this model have been commonly discussed in the context of typical and atypical development, and roles in behavior (Harvey et al., 2004; Friedman et al., 2011; Karasinski, 2015; Roelofs et al., 2015; Blair, 2016). The processes are: withholding a dominant or highly practiced response [“inhibition” (inhibit)]; the regular monitoring and revising of working memory content [“updating” (update)]; and changing flexibly between tasks and mental sets [“switching” (switch)] (Nee et al., 2013). The most recent incarnation of the integrative model identifies an underlying commonality (“common executive”)—assumed to contribute to all executive processes. It has been argued, to be virtually indistinguishable from inhibition—alongside separable switching and updating processes, which rely on common EF and corresponding unique components (Friedman et al., 2008, 2011; Miyake and Friedman, 2012).

Critically then, there is a currently open question about which executive processes can be viewed as truly separable, and exactly how these are related to each other. This question is fundamentally important for understanding the nature of executive dysfunction in atypically developing populations and its relationship to behavior. For example, taking switching as a purported separable executive process, it has been argued that switching specific demands, which require flexibility, oppose goal maintenance in the face of distractions, which are demands that have been attributed to common executive (Goschke, 2000; Dreisbach and Goschke, 2004; Blackwell et al., 2014). Indeed, individual differences in different executive processes have been associated in opposite directions, with attention problems and self-regulatory behaviors (Friedman et al., 2007, 2011; Young et al., 2009). Yet much work on atypically developing populations has tended to take a perspective driven by the measures available, with relatively little attention to underlying structure. Therefore, this approach has often not allowed measure-related and process-related effects to be clearly distinguished (e.g., Van Eylen et al., 2011). Better understanding of how EF processes can be separated is thus required to drive productive research on how these processes can be impaired and the effects of such impairment. One way to further this understanding is with examination of neural constituents of EF.

Since its initial description, the integrative EF model has been applied to child samples in several EF test performance based studies (Hughes, 1998; Lehto et al., 2003; Davidson et al., 2006; Agostino et al., 2010; Rose et al., 2011; Lee et al., 2013). Early results from both exploratory and confirmatory factor analyses showed that—as in adults—there are three inter-related executive processes in children aged 8–13 years (Lehto et al., 2003). However, in subsequent studies switching and updating have not always been distinguishable (Huizinga et al., 2006; St. Clair-Thompson and Gathercole, 2006; van der Sluis et al., 2007; Wiebe et al., 2011; Miller et al., 2012; Usai et al., 2014). Thus, even applying closely equivalent approaches, the question of how applicable the integrative model is to the developing brain remains to be resolved. It is important to note that these studies have applied a range of different measures to examine EF in children, which could contribute to the inconsistent findings. A neural functional approach that includes multiple measurement approaches can help to resolve this inconsistency.

In adults, attempts to examine the structure of EF in a neural context have generally provided support for the integrative model. For example, application of a computational neural network model has provided support for common EF and a switching specific process (Herd et al., 2014). Further, meta-analyses of fMRI data have discriminated patterns of activation across putatively separable executive processes (Lenartowicz et al., 2010). Yet, have still identified common activation indicative of an overarching EF network (Niendam et al., 2012). However, even in adults, attempts to examine the neural constituents of multiple executive processes in the same meta-analysis (Buchsbaum et al., 2005; Derrfuss et al., 2005) have been limited by use of a single task to tap each process. Thus, making it impossible to distinguish between EF process-related and EF task-related findings (Nee et al., 2013).

In children on the other hand, neuroimaging work has generally focused on the emergence and maturation of specific executive processes in children. The development of inhibition, switching and updating (in the broader context of WM) has been examined separately (Kwon et al., 2002; Durston et al., 2006; Morton et al., 2009; Satterthwaite et al., 2013; Kharitonova et al., 2015; Murphy et al., 2016). When assessed collectively, the evidence suggests that from an integrative model perspective, we might expect common executive, switching, and updating to show distinguishable developmental trajectories. Indeed, previous fMRI examinations have found age-related activation changes, pertaining to inhibition, switching and updating, respectively, during childhood and adolescence (Kwon et al., 2002; Durston et al., 2006; Morton et al., 2009).

There is a clear lack of meta-analytic investigation using neuroimaging data pertinent to EF in typical children. Many such analyses have incorporated both children and adults in a single sample and have tended to focus on clinical evaluation, particularly those relevant to ADHD, as reported in e.g., (Dickstein et al., 2006; Cortese et al., 2012; Hart et al., 2013). In addition, existing adult and/or child fMRI meta-analyses have tended to take a process specific or task specific approach rather than attempting to address how multiple executive processes are related to one another (e.g., Criaud and Boulinguez, 2013). Whole brain analyses also need to be utilized, as much of the literature considers a region of interest approach e.g., the insula (Chang et al., 2013), or right ventrolateral prefrontal cortex (Levy and Wagner, 2011). Only one meta-analytic study, conducted by Houdé et al. (2010), has reviewed the three executive processes considered in the integrative EF model, using fMRI data from typical children and adolescents (aged 4–17 years, using an age cut-off of 11.4 years, as this was the midpoint). Houdé et al. found regions of activation similar to those reported in adult samples. Yet, the authors only examined “collective” activity pertaining to inhibition, updating and switching (which from an integrative model perspective could be viewed as common EF). But did not assess activation specific to individual executive processes. Thus, the findings cannot inform on the potential applicability of the integrative EF model to children or the relative commonality vs. dissociation of individual processes.

The present study investigates the structure of EF in children and adolescents, by examining fMRI activation during EF task performance. The executive processes of interest include inhibition, updating, and switching, as emphasized by Miyake's integrative model. Further, an additional variable representing the unitary executive process (“common executive”), which amalgamates all three executive processes of interest, is considered. BrainMap GingerALE software (version 2.3) was used. In line with Miyake and Friedman's integrative model and the hierarchical model of EF development proposed by Garon et al. (2008), we hypothesize that activity relating to inhibition and common executive will largely indicate shared activation. This finding would provide support for inhibition and common executive processes being indistinguishable at a neural level. On the other hand, we hypothesize that significant non-shared activation will become apparent when common executive is compared to switching and updating, indicating the presence of switching-specific and updating-specific components of EF in children.

## Methods

### Design

Papers relating to inhibition, switching, and updating were identified. Following this, Activation-Likelihood Estimation (ALE) maps were produced to examine the location of brain activation during inhibition, switching, and updating task engagement in the whole sample group (aged 6–18 years). Similarly to the study by Houdé et al. (2010), comparable ALE maps were also created from studies comprising only children (6–12 years; “child” group). Separate maps for each of the executive processes were created and a “common executive” map comprised shared activation across tasks tapping the individual executive processes. Areas of significant overlap and differentiation in these maps were compared to examine neural integration vs. distinction of the EF processes.

### Study selection

Literature searches were conducted in Web of Science, PubMed and PsycINFO between 23rd October 2014 and 24th April 2015. Keyword searches comprised the following terms combined with AND operators: 1. fMRI OR “functional magnetic resonance imaging”; 2. child^*^; 3. inhibition OR stroop OR “flanker task” OR switching OR updating etc. A full list of the terms used is reported in Table 1. Multiple terms were used for each executive process of interest. Where specific EF tasks with commonly used names were identified, these names were added to the search, e.g., a study employing a Stroop task did not have to include the key word “inhibition” to be identified. Notably, more such specific tasks were identified for inhibition (see Table 1). Some tests sometimes labeled as EF tests—such as WM span tasks—measure WM capacity, which we and others consider to be the passive storage of information in short-term memory, a different construct to WM updating (Lehto, 1996; Miyake et al., 2000; Chein et al., 2011). Such tests were therefore excluded from the present meta-analysis.

**Table 1:** **List of terms used in database searches**.

**Search terms**	
fMRI OR “functional magnetic resonance imaging” AND child^*^ AND.…	Inhibition Go-No/Go Stroop Anti-saccade Simon Flanker “Stop Task” Stop-signal “Inhibition of an orientating response” Switching Shifting Cognitive flexibility Flexibility “Task switching” “Set shifting” “Task shifting” “Set switching” Updating “Working memory updating” “n back”

Initial inclusion criteria were typically developing child participants (aged 6–18 years) engaging with an inhibition, switching or updating task during fMRI acquisition. Consequently, 195 papers were retrieved from these searches. Typical development was defined as having had no prior diagnosis of a psychological problem. Thus, children could be deemed typically developing despite their suggested risk of a psychiatric disorder based on for example, expression of a genetic polymorphism variant or score on a clinical scale using “at risk” cut-offs (e.g., Mechelli et al., 2009; van 't Ent et al., 2009). Following this, authors who did not report activations in standard stereotactic coordinate space (Talairach or Montreal Neurological Institute) were contacted and asked to forward coordinate activations if possible. Thus, unpublished data were included in the analysis. If appropriate data were not received by 30th April 2015, the paper was excluded. Authors were also approached if only between groups (higher-level) comparisons were reported. Or if activations isolating the executive process(es) of interest were not addressed, i.e., they had to report a contrast between an executive demand condition and a matched comparison condition that did not apply the executive demand. Further, if papers only provided activation data recorded during the pre-or post-stimuli intervals or if the contrasts were indicative of successful vs. failed responses and vice versa. Once these parameters were applied, 90 papers remained. Region-of-interest (ROI) analyses were excluded to prevent an activation bias (Poldrack, 2007; Kriegeskorte et al., 2009). Some papers incorporated multiple experiments, either within or across the three executive processes. However, if needed, further contact with the authors was made to ensure that data from one group of participants during an EF task reported in multiple papers or at multiple time points, was not duplicated. On the other hand, if the same participants completed more than one EF task, the data from these tasks was included. Consequently, 49 papers endured, but with 53 experiments. Of these studies, six included eight datasets that have never been published before. Further to the database search, the reference lists from all applicable papers were also examined to identify potential additions to the meta-analysis, however, this resulted in no additional papers.

The final dataset included 1,177 participants with a mean sample age more than 6 years and <18 years (Table 2). The whole sample dataset incorporated 573 activation foci, and the child group incorporated 549 participants across 29 experiments, containing 317 activation foci. The cut-off for the child group was based on previous research indicating that executive processes tend to be relatively mature by the age of 12, yet not “fully established” (e.g., Anderson, 2002). A demographic summary of each study including study name, participant age, number of participants, EF task used, stimuli, contrast and number of foci, is outlined in Table 2.

**Table 2:** **List of studies included in the meta-analysis**.

	**Study**	**Task**	**Mean Age (*sd*) *r***	***n***	**Contrast**	**Foci**
Inhibition	Fan et al., 2014	Number stroop	11.2 (2.9)	23	incongru > congru	1
	Liu et al., 2008	Color stroop	14.3 (3.3)	10	incongru > congru	18
	Posner et al., 2011	Number stroop	13.4 (1.2)	15	number blocks vs. neutral blocks	5
	van 't Ent et al., 2009	Color stroop	15.17 (1.45)	18	incongru > congru	19
	Anderson et al., 2005	Shape GNG	13.63 (0.88)	46	no-go > go	2
	Bennett et al., 2009	Letter GNG	12	11	no-go > go	8
	Durston et al., 2003	Picture GNG	8.68 (1.51)	7	no-go > go	8
	Heitzeg et al., 2014	Letter GNG	10.9 (1.1) *r* = 9.4–12.9 (baseline)	19	no-go > go	6
	Iannaccone et al., 2015	Arrow non-spatial GNG	14.82 (1.24) *r* = 12–16	18	no-go > go	17
	Lei et al., 2012	Letter GNG	11.5 (1.9)	22	no-go > go	14
	Mechelli et al., 2009	Picture GNG	11.32 (.67)	102	no-go > go	8
	Nosarti et al., 2006	Arrow non-spatial GNG	17.2 (1.1)	14	no-go - odd trials	10
	Querne et al., 2008	Letter GNG	10 (1.1) *r* = 8.2–11.6	10	no-go > go	14
	Sheinkopf et al., 2009	Picture GNG	*r* = 8–9	12	no-go > go	4
	Simmonds et al., 2007	Picture GNG	10.6 (1.5) *r* = 8–12	30	no-go > go	10
	Siniatchkin et al., 2012	Picture GNG	9.1 (4.1) *r* = 7–13	14	no-go > go	12
	Singh et al., 2010	Letter GNG	14.3 (2.33)	22	no-go > go	2
	Suskauer et al., 2008	Picture GNG	10.8 (1.3)	25	no-go > go	7
	Tamm et al., 2004	Letter GNG	15.58 (0.79) *r* = 14–16	12	no-go > go (a vs. b)	3
	Fitzgerald et al., 2008	Shape A-S	11.5 (1.8) *r* = 8–14	11	Anti-correct vs. pro-correct	12
	Christakou et al., 2009	Simon task	r = 10-17	36	incongru > congru	3
	Halari et al., 2009	Simon task	16.3 (1.1)	21	incongru > congru	6
	Rodehacke et al., 2014	Simon task	14.6 (0.3) *r* = 13.7–15.5	185	incongru > congru	14
	Rubia et al., 2006	Simon task	15 *r* = 10–17	29	incongru > congru	5
	Sheridan et al., 2014	Simon task	8.1 (1.66) *r* = 5.7–10.7	33	incongru > congru	7
	Bhaijiwala et al., 2014	Letter Stop task	15.4 (1.7) *r* = 8–19	12	stop > go	4
	Cubillo et al., 2014a	Arrow Stop task	13.9 (1.7) *r* = 10–17	29	stop > go	9
	Ware et al., 2015	Letter Stop task	15.09 (1.51) *r* = 13–16	21	stop > baseline (all stop coords)	7
	de Kieviet et al., 2014	Flanker task	8.7 (0.5)	47	incongru > congru/neutral	2
	Vaidya et al., 2005	Flanker task	9.2 (1.3)	10	incongru > neutral	4
	van 't Ent et al., 2009	Flanker task	15.17 (1.45)	18	incongru > congru	20
Switching	Christakou et al., 2009	Spatial switching	*r* = 10-17	36	switch > repeat	4
	Dibbets et al., 2006	Picture switching	6.83 (.53)	7	switch > nonswitch	13
	Halari et al., 2009	Spatial switching	16.3 (1.1)	21	switch > repeat	8
	Rodehacke et al., 2014	Arrow switching	14.6 (0.3) *r* = 13.7–15.5	185	switch > repeat	19
	Rubia et al., 2006	Spatial switching	15 *r* = 10–17	29	switch > repeat	5
	Wendelken et al., 2012	Picture switching	10.56 *r* = 8–13	20	switch > repeat	9
Updating	Beneventi et al., 2010b	Letter n back	13.5 (0.5)	14	1 /2 back > 0 back	13
	Beneventi et al., 2010a	Phoneme n back	13.5 (0.5)	13	2 back > 0 back	13
	Bennett et al., 2013	Number n back	12.6 (0.2)	11	2 back > 1 back	17
	Chang et al., 2004	Visuospatial n back	14.4 (3.2)	10	2 back > 0 back/control	6
	Ciesielski et al., 2006	Categorical n back	6.1 (0.55) *r* = 5.11–6.6 & 10.1 (0.45) *r* = 9.1–10.5	17	2 back > 0/1 back	26
	Cservenka et al., 2012	Letter n back	14.18 (0.7)	16	2 back > 0 back	3
	Cubillo et al., 2014b	Letter n back	13.7 (2.4) *r* = 10–17	20	1 b > 0 b, 2 b > 0 b, 3 b > 0 b	20
	Li et al., 2014	Categorical n back	10.9 (2.7) *r* = 8–16	27	2 back > 0/1 back	3
	Massat et al., 2012	Number n back	10.05 (1.28)	14	2 back > 0 back	17
	Malisza et al., 2005	Spatial n back	*r* = 7–12 (1)	8	1 back > 0 back	13
	Nagel et al., 2013	Spatial & letter n back	13.11 (1.78) r = 10-16	67	2 back > 0 back	21
	Nelson et al., 2000	Visuospatial n back	*r* = 8–11.7	9	2/1 back > 0 back	10
	Robinson et al., 2014	Letter n back	12.9 (2.78)	15	2 back > 0 back, 3 back > 0 back	18
	Thomas et al., 1999	Spatial n back	9.8 *r* = 8-10	6	2/1 back > 0 back (individually assessed)	7
	Vuontela et al., 2009	Location & Color n backs	12.2 *r* = 11–13	8	L2 back > L0 back & C2 back > C0 back	42
	Vuontela et al., 2013	Face 1 back & scene 1 back	9.06 *r* = 7–11	16	Face 1 back > rest & Scene 1 back > rest	18
	Yu et al., 2011	Categorical n back	11.3 (1)	15	2 back > basal stimulus	7

*Main study demographics are outlined: EF task administered, mean age (in years), sample size (n), the fMRI contrasts of interest and the number of foci of significant activation associated with the contrast*.

*Standard deviation is reported in brackets; r, range; congru, congruent; incongru, incongruent; GNG, Go-No/Go; b, back (e.g., 1 b); L, letter (e.g., L2 back); C, color (e.g., C0 back); where “and” is reported, two separate contrasts were included in the analysis*.

^*^ For references of meta-analysis papers, see Reference Section.

### Analysis

#### Activation-likelihood estimation (ALE)

BrainMap GingerALE software (version 2.3) was used to perform an ALE meta-analysis. Analyses were conducted based on Montreal Neurological Institute (MNI) coordinates and coordinates originally published in Talairach and Tournoux (1988) stereotactic-space were converted to MNI using the Lancaster transformation (Lancaster et al., 2007). ALE is a coordinate-based technique based on voxel-wise foci of significant activation across the included studies. Activation foci from separate studies are mapped in a common stereotactic space to highlight consistent conjunction. The ALE method calculates the number of activation peaks across each brain region and compares this to a uniform activation distribution representative of a null hypothesis (which is when there are not enough peaks in a voxel to indicate that at least one peak truly activates in that voxel; Wager et al., 2007). The activation foci are then treated as 3D Gaussian probability distributions and incorporated into a modeled activation map for each study. Data are filtered through a Gaussian kernel, which is sensitive to each study's sample size (Eickhoff et al., 2009, 2011). It is important to note that while the ALE method considers conjunctive activation, a study with more participants can contribute more to the overall results (Wager et al., 2007). The ALE statistic means that within a given voxel, at least one or more significantly activated peaks apply (Turkeltaub et al., 2002). In the present study, the random sampling was subjected to 5,000 iterations to compute a null distribution. This was then used to compare with voxel-wise ALE values to calculate statistical parameters (Nee et al., 2013). The ALE maps were thresholded at *p* < 0.05 corrected for multiple comparisons by false discovery rate (FDR; Laird et al., 2005) and a cluster threshold of 100 mm^3^ (Hill et al., 2014) was employed in the first-level analyses.

#### First-level analyses

**Figure 1:**
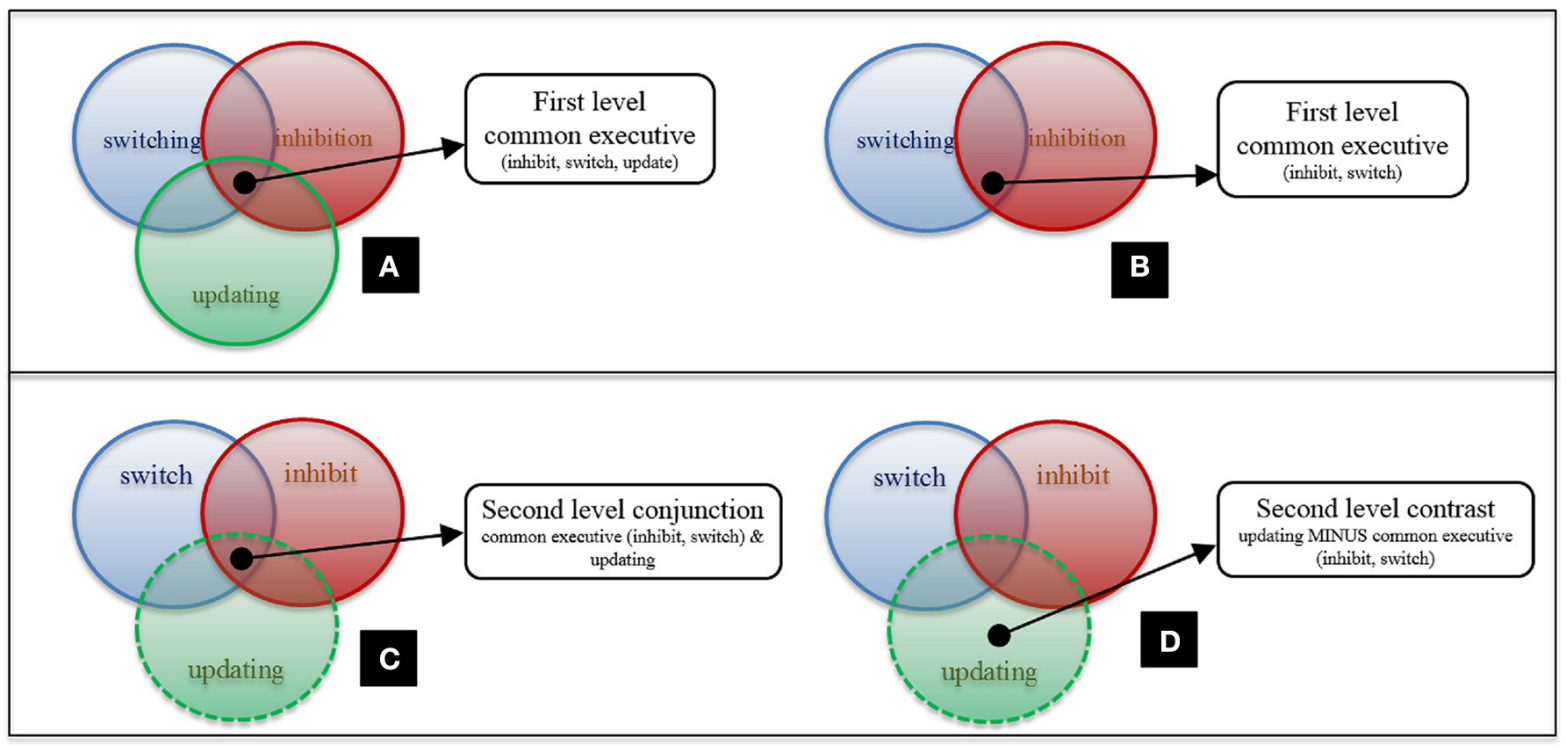
**First and second-level analysis design. (A)** First-level Common Executive (inhibit, update, switch); **(B)** First-level Common Executive (inhibit, switch); **(C)** Second-level Conjunction Analysis for Common Executive (inhibit, switch) and Updating; **(D)** Second-level Contrast Analysis for Common Executive (inhibit, switch) and Updating. N.B. There are statistical differences between **(A,C)**.

First-level analyses on common executive (shared activation across tasks tapping inhibition, switching, and updating executive processes; Figure 1A) and each specific putative executive process (inhibition, updating, and switching) were conducted. First-level analyses describe clusters that pass the applied threshold for significant conjunctive activation across these groups of studies. These analyses were computed for both the whole sample and the child group separately.

#### Second-level analyses

Second-level analyses compare two first-level analyses, examining significant similarities and differences in activation. Second-level conjunctions reveal significant shared activation between two ALE maps. While second-level contrasts reveal significant non-shared activation between two ALE maps, by subtracting one ALE map from the other. To achieve these analyses whilst controlling for different sample sizes across studies, simulated data is created by pooling datasets and randomly dividing them into two groups of equal size. These groups are also equivalent to the original data sets' sizes. The ALE images from the new datasets are then compared to each other; and resultant conjunctions/contrasts are compared to those in the true data. Following many permutations, a voxel-wise *p*-value image is created and transformed to a z score to indicate significance (Eickhoff et al., 2011).

To examine the distinction between each executive process and common executive, the shared and non-shared activation between these processes was investigated. Since analyses pool data across studies, including the same study in common executive and process specific maps for second-level analyses, would introduce a bias toward significant conjunction. Thus, at the second level, analyses were conducted so as to prevent any individual study being included in two first level maps being compared. For example, in second-level analyses for updating and common executive, the “updating” map was compared to a “common executive (inhibit, switch)” map (Figure 1B). Conjunction analyses to assess activation pertaining to the executive component of the executive process of interest—in this case, updating—were conducted (Figure 1C). As were contrast analyses which examined updating-specific activity (Figure 1D). Corresponding analyses were also administered for switching and inhibition. This technical necessity is thus consistent with our theoretical stance. Here, the common executive construct is defined as a system drawn on by all other executive processes (including the three specific processes focused on here but also others that are not the present focus). Thus, we are working from the assumption that shared activation across two; or three; or more individual executive processes should be equally capable of identifying the common executive component at a neural level.

#### Control analyses

Further second-level analyses, which we will refer to as “control analyses” were conducted to examine the putative similarities and differences between common executive, switching, and updating. The control analyses were designed to control for the lower number of switching studies in the data set. These conjunction and contrast analyses incorporated subsamples of common executive, which comprised inhibition, switching and updating datasets with ~58 foci each (to match the maximum number of switching foci obtained). These were then compared with subsamples of each specific executive process (again with ~58 foci each). Again, to reduce bias, each specific executive process subsample contained different studies from their comparative subsample in the common executive dataset. The foci included in each common executive dataset were chosen at random, while ensuring that approximately equal numbers of foci from each EF task were represented. Four different subsample datasets were computed for common executive and updating and thus, four control analyses were conducted. As there is only one switching dataset, we created four subsample datasets with inhibition and updating only (~58 foci each) and contrasted these with the switching dataset, resulting in four separate analyses. Thus, for the examination of updating vs. common executive activation, these control analyses included a common executive map derived from studies that included inhibition, switching, and updating tasks. The analyses therefore allowed some verification of the assumption that common executive activity can be isolated from shared activation across tasks tapping two; three or more executive processes.

## Results

### Common executive and inhibition

#### First-level common executive analyses

**Figure 2:**
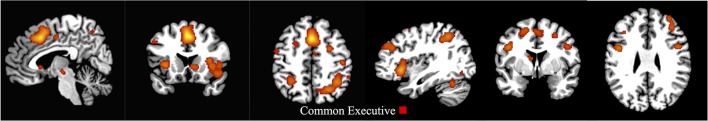
**First-level analysis for common executive in the child/adolescent group (*x* = 5, *y* = 17, *z* = 47; *x* = 113, *y* = 75, *z* = 58)**. ALE maps showing the significant activation clusters of common executive for the child/adolescent sample (29 clusters).

The first-level ALE map for common executive in the whole sample demonstrated shared activation in 29 clusters, with the largest activation in the right and left middle and superior frontal gyri and the right and left supplementary motor area. Right parietal regions, such as the supramarginal gyrus, the inferior, and superior parietal gyri including the intraparietal sulcus (IPS), the precuneus, and the angular gyrus, as well as the left inferior and superior parietal gyri were activated. Activation was also present in the anterior insular cortex (AIC; Figure 2 and Supplementary Materials Section A).

**Figure 3:**
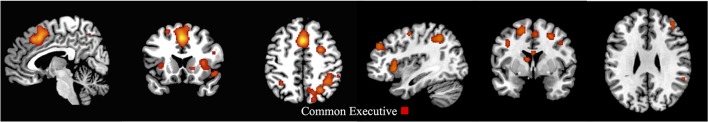
**First-level analyses for common executive in the child group (*x* = 5, *y* = 17, *z* = 47; *x* = 113, *y* = 75, *z* = 58)**. ALE maps showing the significant brain activation for common executive in the child group (30 clusters).

The common executive first-level ALE map for the child group showed 30 clusters, and like the child/adolescent group, the largest cluster extended between the right and left supplementary motor area, the right and left middle cingulum, and the right and left superior and medial frontal gyri. The same right parietal regions as the whole sample were activated, as well as the right middle frontal and precentral gyri (Figure 3 and Supplementary Materials Section B).

#### First-level inhibition analyses

**Figure 4:**
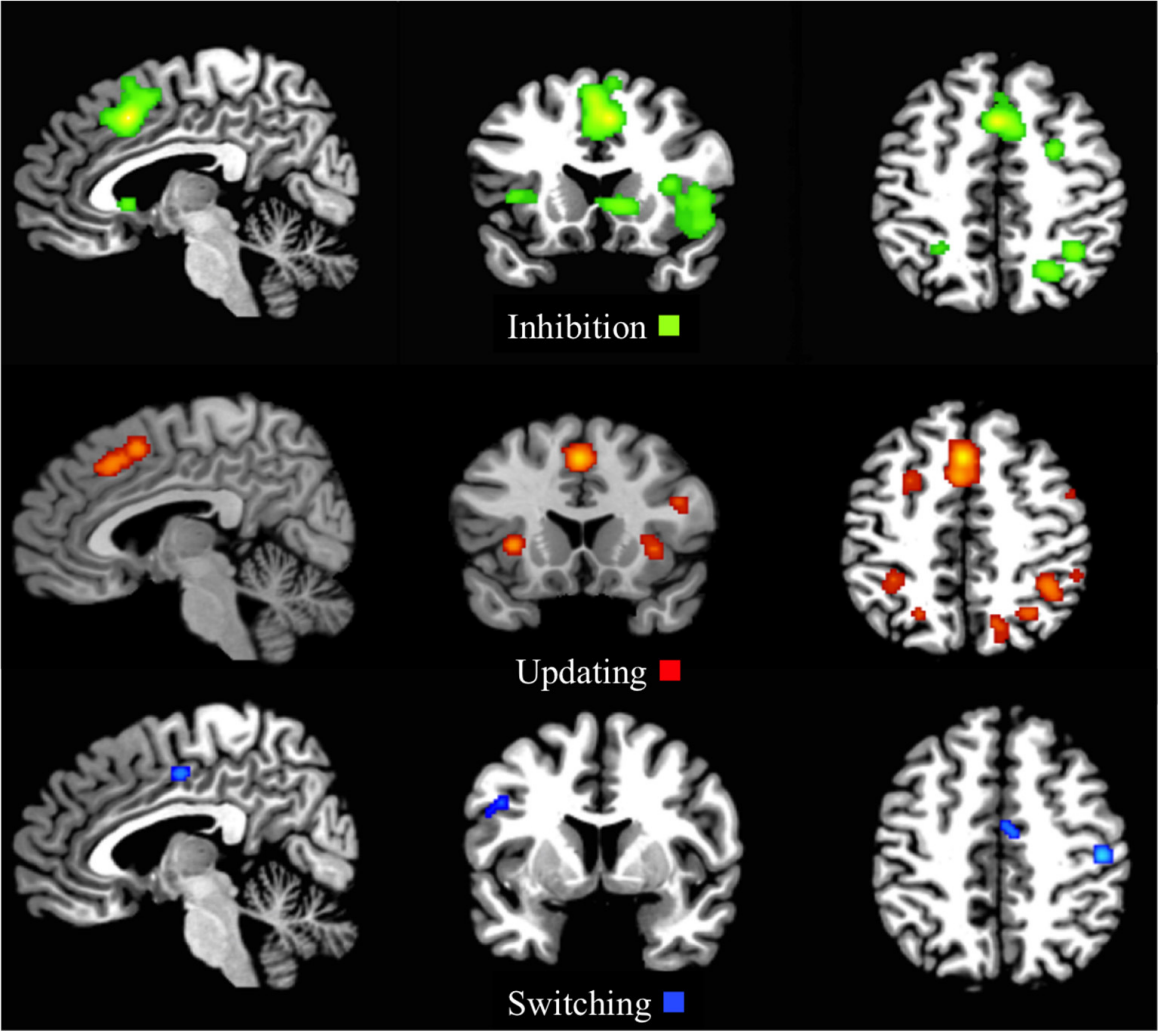
**First-level analyses for inhibition (*x* = 5, *y* = 17, *z* = 47), updating (*x* = 5, *y* = 17, *z* = 47), and switching (*x* = 5, *y* = 5, *z* = 46) for the child/adolescent group**. ALE maps reveal the significant activation clusters of Inhibition (20 clusters), updating (25 clusters), and switching (4 clusters) in the child/adolescent group.

The whole sample ALE map for the inhibition first-level analysis indicated 20 activation clusters, with the largest clusters residing in the right and left superior and medial frontal gyrus and right and left supplementary motor areas. Large clusters were also located in the right inferior frontal gyrus extending to the right AIC and right superior temporal pole, as well as the right parietal regions, including the IPS (Figure 4 and Supplementary Materials Section A).

**Figure 5:**
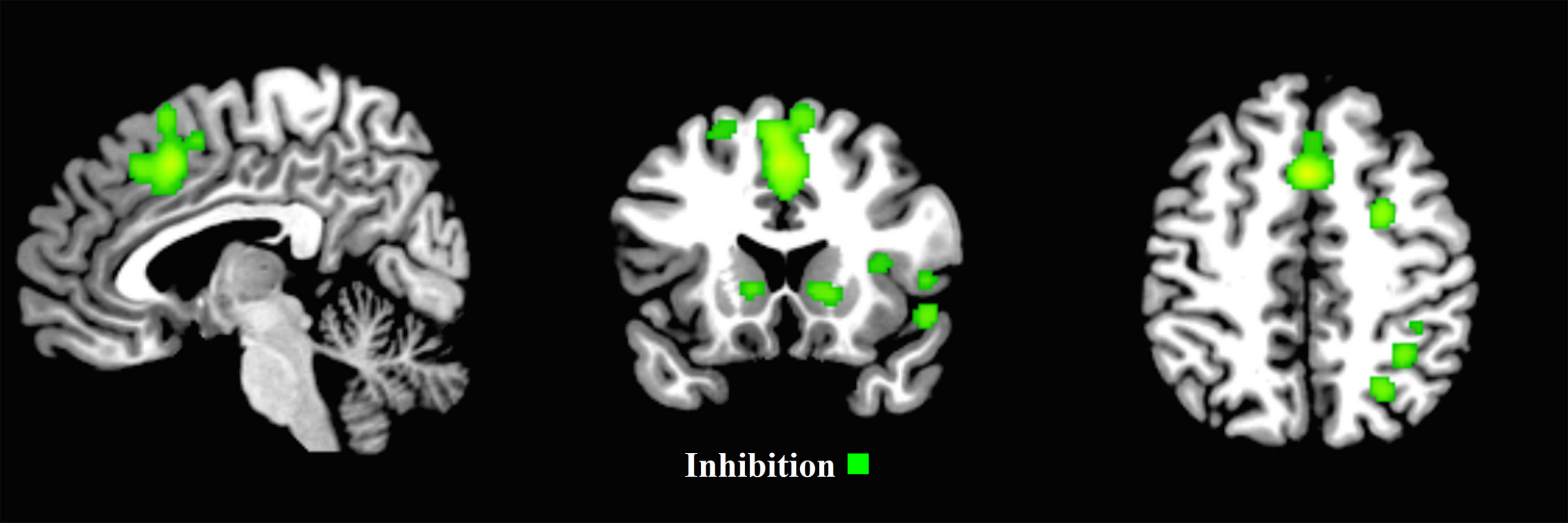
**First-level analyses for inhibition for the child group (*x* = 5, *y* = 17, *z* = 47)**. ALE maps reveal the significant activation clusters of inhibition for the child group (18 clusters).

The ALE inhibition first-level map for the child group revealed 18 activation clusters. The main patterns of activation were evident in the frontal areas, including the right frontal eye fields (FEF), with clusters extending from the left and right supplementary motor areas, through the left and right medial frontal gyrus, to the left and right middle cingulum (Figure 5 and Supplementary Materials Section B).

#### Second-level analyses

The conjunction analysis for common executive (update, switch) compared with inhibition revealed 10 shared clusters in the whole sample and 5 in the child group. The areas with the most significant activation in the whole sample included the left medial and superior frontal gyri; bilateral areas of the insula and parietal areas; and right sided activation in the precentral gyrus, claustrum, and precuneus. Whereas, the areas with significant activation in the child group resided bilaterally in the medial frontal gyri and right sided activation in the cingulate gyrus, claustrum, the inferior parietal lobe, and precuneus. However, the contrast analysis did not identify any significant differences for either sample. This is consistent with the view that inhibition is not separable from a common executive capacity (Supplementary Table C and Supplementary Figure 1, and Supplementary Table D and Supplementary Figure 2).

### Common executive and updating

#### First-level updating analysis

The first-level ALE map for updating displayed 25 clusters, with the main activation demonstrated in right and left frontal medial gyrus, including the FEF, extending to the supplementary motor areas and middle cingulum extending to the anterior cingulate cortex (ACC). Other clusters included extensions from the right pars opercularis to the right precentral gyrus, the left and right inferior parietal lobule (with the right sided activation spreading to the supramarginal gyrus and IPS), the right and left middle frontal gyri to the superior frontal gyri and the right and left insula (Figure 4 and Supplementary Materials Section A).

#### Second-level analyses

**Figure 6:**
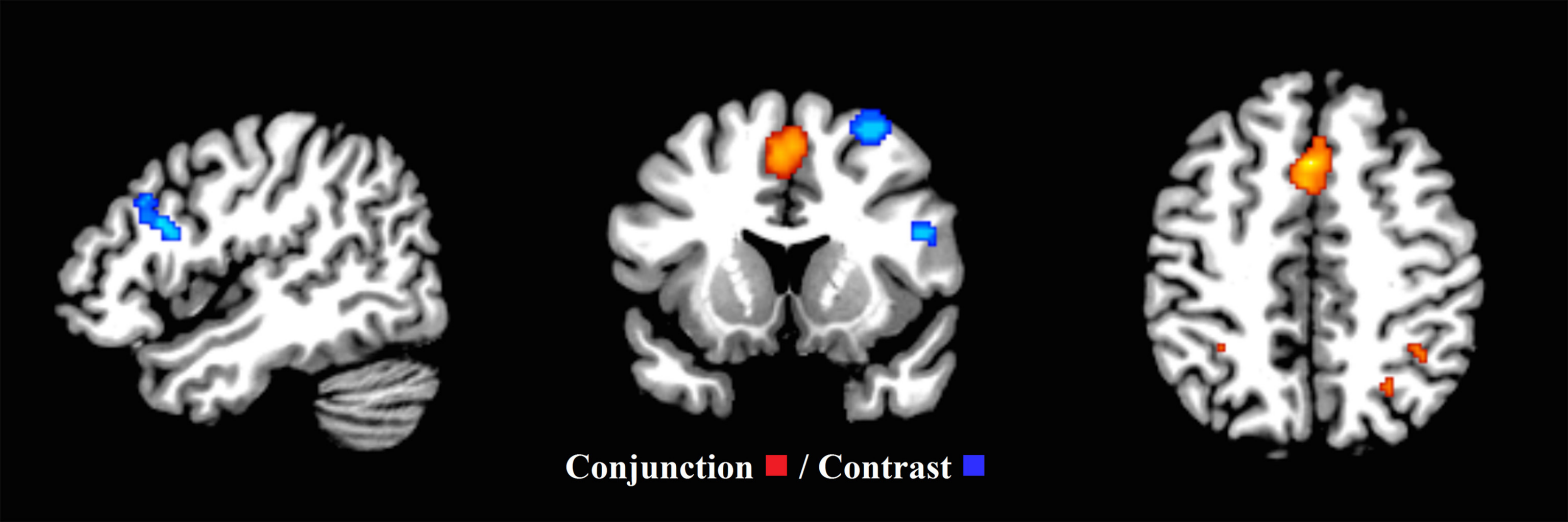
**Common executive (inhibit, switch) and updating (*x* = 47, *y* = 13, *z* = 46)**. Significant conjunction and contrast analysis results for common executive (inhibit, switch) and updating. Regions of significant conjunction (eight clusters—red) and contrast (four clusters—blue) are displayed. The clusters indicating non-shared activation were found when the common executive (inhibit, switch) dataset was subtracted from the updating dataset.

Examining the common executive component of updating, the second-level conjunction analysis produced 8 clusters in the whole sample (ranging between 40 and 2,576 mm^3^ in size). These mainly resided in the left and right superior frontal gyrus continuing to the medial frontal gyrus and extending to the right cingulum and right supplementary motor area, the left and right insula and the right inferior and superior parietal lobes (Figure 6 and Supplementary Materials Section E). The second-level conjunction analysis for the child group resulted in six clusters, residing bilaterally in the medial frontal gyrus, the right cingulate gyrus, claustrum, and right parietal areas (Supplementary Table F and Supplementary Figure 3).

To examine a putative “updating specific” component of updating, the second level contrast analysis revealed four clusters (ranging between 144 and 1,136 mm^3^). These clusters were located in the right middle and superior frontal gyri, as well as the pars triangularis and pars opercularis in the right inferior frontal gyrus, and the left and right cerebellar crus I and II (Figure 6 and Supplementary Materials Section E). However, the second-level contrast analysis revealed no significant clusters in the child group.

### Control analyses

Four second-level control analyses were conducted using foci-matched common executive and updating datasets. This provided a matched point of comparison to the switching analyses. And tested whether the pattern of significant non-shared common executive vs. updating activity exists when the common executive map includes updating tests. Two of the analyses identified contrast clusters when common executive was subtracted from updating. The first found one contrast cluster (216 mm^3^) extending between the right inferior and superior parietal lobe. The second found two clusters, with the largest (304 mm^3^) residing between the right middle frontal gyrus and the right precentral gyrus. While the smaller (104 mm^3^) extended between the left cerebral crus I and left cerebellar lobule VI (Supplementary Table H and Supplementary Figure 4). These findings demonstrate that although the power of the analysis has been compromised, due to the lower number of foci included, updating-specific activity is still apparent.

### Common executive and switching

#### First-level switching analysis

The first-level analysis for switching resulted in four activation clusters. The largest cluster was located in the right postcentral gyrus in the parietal lobe, with other clusters residing in the right middle cingulum extending to the ACC, the left precentral gyrus extending to the pars opercularis in the inferior frontal gyrus and the left lingual gyrus spreading to the left calcarine (Figure 4 and Supplementary Materials Section A).

#### Second-level analyses

**Figure 7:**
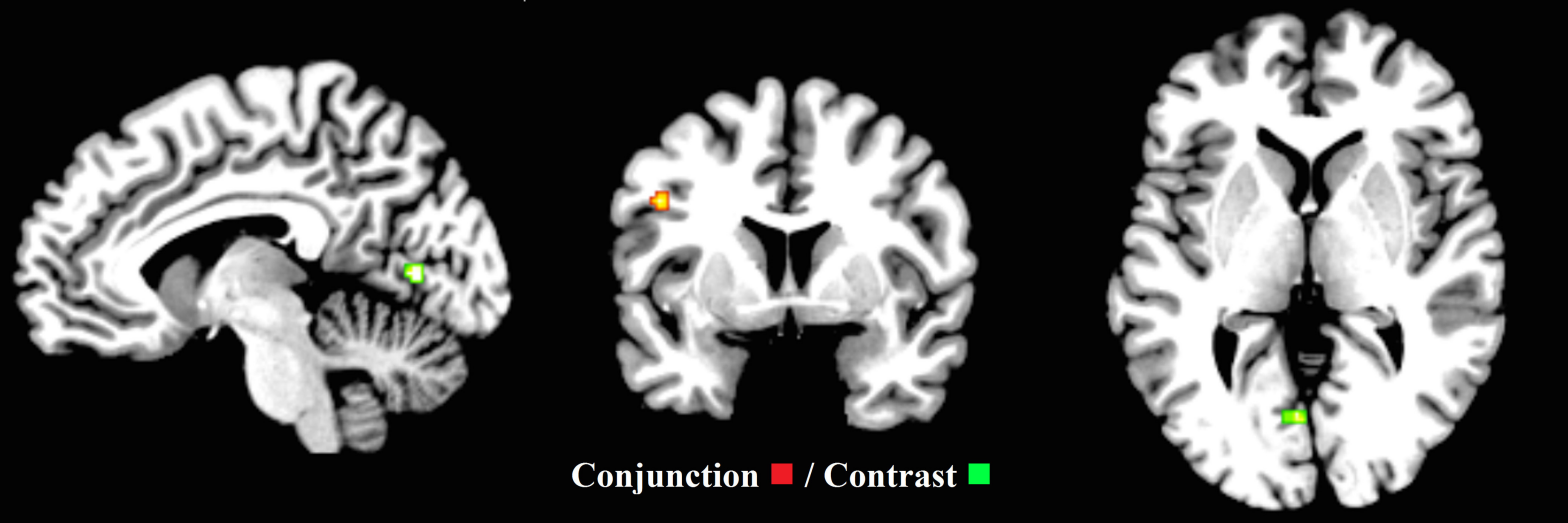
**Common executive (inhibit, update) and switching (*x* = −7, *y* = 4, *z* = 1)**. ALE maps demonstrate the significant conjunction (one cluster—red) and contrast activation (one cluster—green) for common executive (inhibit, update) and switching. The contrast cluster was produced when the common executive (inhibit, update) dataset was subtracted from the switching dataset.

Furthermore, to examine the putative common executive component of switching, the second-level conjunction analysis revealed one cluster (88 mm^3^) extending between the left precentral gyrus and the left frontal inferior operculum. To examine the putative “switching-specific” component of switching, the second level contrast analysis revealed one cluster (192 mm^3^) in the left lingual gyrus extending to the left calcarine (Figure 7 and Supplementary Materials Section G). These findings support the view that common executive and switching-specific components of switching may be separable at a neural level. Conjunction and contrast analyses were conducted for the child group, however, due to the low number of studies, no clusters pertaining to shared or non-shared activation were revealed.

### Control analyses

Finally, four control analyses were also generated for the equivalent switching data, however, no significant differences were found in the contrast analyses.

## Discussion

Here, an ALE meta-analysis investigated overlap and differentiation in neural activation pertaining to inhibition, switching, updating and the putative unitary “common executive” capacity in children under the age of 18. Results suggest an overlapping yet distinct neural structure of executive function, as previously reported in adults (Collette et al., 2006). No inhibition-specific neural correlates unrelated to the common executive were identified in either the whole sample (child/adolescent) or in the child only group. Further, when updating and switching were compared to the unitary common executive, shared neural activation was demonstrated, pointing toward common executive components of switching and updating. However, such comparisons also revealed non-shared neural activation linked to updating and switching, pointing toward separable updating-specific, and switching-specific entities in the whole sample. Specifically focusing on the child group relied on analyses with less power. Nevertheless, it is important that no evidence could be provided to support updating or switching-specific separable entities in the child group, despite substantial data being available to examine this possibility for updating.

When common executive activity was isolated, it revealed significant bilateral activation in fronto-parietal areas and regions of the supplementary motor area in the whole sample group. The corresponding analysis limited to the child group demonstrated significant activity in largely the same areas. These results are in line with previous findings, which show activity in these areas during EF tasks throughout the child and adolescent years (Chambers et al., 2009). Further, activation in these regions has also been linked to conjunctive activity across inhibition, switching and updating tasks in adults aged 18–60 years (Niendam et al., 2012). This is consistent with the EF “fronto-parietal flexible hub” theory posited by Cole et al. (2013), which is based on functional neural connections engaged during EF. Previous meta-analyses assessing EF activation have also generated results indicative of shared neural activity. One such analysis, conducted by Derrfuss et al. (2005), assessed the role of the inferior frontal junction (IFJ) during switching and Stroop task performance. Both analyses showed concurrence of activation in the IFJ, yielding support for an overlap of shared resources between the two executive process paradigms. Since the IFJ is part of the fronto-cingulo-parietal network, this study provides further support for the present results. Furthermore, as the study by Derrfuss et al. examines adult data, our results suggest a similar EF structure may be apparent in children.

In the present study, common executive activity coincided with activity linked to inhibition—isolated from shared activation across only inhibition tasks—in both the whole sample, and the child only group. However, for activity linked to inhibition tasks, larger clusters of right parietal activity were evident in the whole sample relative to the child group. Although our analyses could not make direct statistical comparisons between the two sample groups, these findings are generally consistent with progressive age-related increases in parietal activation during inhibition engagement (Rubia et al., 2006; Neufang et al., 2008). This is also consistent with further evidence reporting a right laterality effect in adolescents compared to children (Houdé et al., 2010). In line with the apparent similarities across common executive and inhibition related activation maps, our findings demonstrated areas of statistically significant shared activation across common executive and inhibition. Although, direct comparison between activation pertaining to inhibition and common executive has not been the focus, many previous studies have reported corresponding areas of activation for these constructs in child, adolescent and adult samples (Wager et al., 2005; Velanova et al., 2008; Niendam et al., 2012; Vara et al., 2014; Lei et al., 2015).

Further, our findings showed of no areas of statistically significant difference across common executive and inhibition in either the whole sample or the child group. This is consistent with our hypothesis and in line with the view that inhibition and common executive are indistinguishable (Friedman et al., 2008, 2011; Miyake and Friedman, 2012). This finding is important because it helps to reconcile some of the previous discrepant findings in the field. For example, previous research on the structure and development of EF suggests a unitary factor representing a common underlying EF process is evident during early-middle childhood. And after this time, distinct executive processes emerge (Tsujimoto et al., 2007; Shing et al., 2010; Brydges et al., 2014; Lerner and Lonigan, 2014). In addition, both Zelazo's cognitive complexity and control theory (Zelazo and Frye, 1998; Zelazo and Muller, 2002) and Munakata's theory (Munakata, 2001) describe EF changes in early childhood as possessing a unitary quality. However, in contrast, Diamond emphasizes the dissociative components of EF during development, yet, she also argues that periods of synthesis of multiple executive processes can occur during times of EF growth spurts in the preschool and early childhood years (Diamond, 2001, 2006). Inhibition is the factor most commonly identified in developmental EF latent variable analysis research, even in very young children, and this may be the first to develop (Garon et al., 2008). Therefore, the present findings suggest that what develops first may be the common component of EF, which is indistinguishable from inhibition during the developmental period. Executive dysfunction at an early age may thus be primarily governed by an inhibition deficit. Due to the apparent strong links with behavior problems, early intervention to improve inhibitory abilities may be key to minimizing the risk of developing clinically-relevant behaviors.

In examining common executive components of updating in children under 18 years, our findings point toward bilateral frontal, right parietal and subcortical activation. Furthermore, updating-specific activation could be distinguished from this pattern in the whole sample group. Updating-specific activity was also frontal but specifically right sided, and further included areas of activation in the cerebellum. Previous work in adults has revealed greater activation in bilateral frontal regions as well as left parietal areas, when updating was compared to switching and inhibition (Collette et al., 2005), pointing toward some correspondence across children and adults in this respect. Previous work in adults has attempted to isolate an updating-specific process from common executive at a neural level using relational analyses between indices derived from performance on cognitive tests; and functional and morphometric indices of brain networks (Reineberg et al., 2015; Smolker et al., 2015). However, relationships between individual differences in updating-specific ability and a resting state functional connectivity network were not demonstrated consistently across all of these indices. It was therefore proposed that updating-specific ability may rely more on a specific area involved in WM and less on connectivity between regions.

Miyake and Friedman (2012) posited that the concept of an updating-specific process, and the abilities it taps, is less clear than the other executive processes. Yet, they have suggested “effective gating of information” and “controlled retrieval from long-term memory” as integral components. This proposal is consistent with work that has examined transformation, substitution—in line with Miyake's effective gating—and retrieval, as updating subsidiary components (Bledowski et al., 2010; Ecker et al., 2010; Zhang et al., 2012). This allows updating to be viewed with respect to performance on measures of WM capacity, which similarly draw on retrieval (Unsworth and Engle, 2008; Ecker et al., 2010). All of the updating tasks included in the present meta-analysis (n back tasks) and the task employed by Reineberg et al. (2015) and Smolker et al. (2015) (keep track), require retrieval (Linares et al., 2016). Thus, since right prefrontal brain regions have been particularly implicated in WM capacity (Prabhakaran et al., 2000; Zhang et al., 2004; Repovs and Baddeley, 2006), the present findings are consistent with the view that the updating specific process identified may rely heavily on neural architecture involved in WM capacity. Previous research has suggested that computerized WM training can increase WM capacity and improve use of WM in everyday life (Spencer-Smith and Klingberg, 2015). However, there has been debate around whether such improvements may transfer to, for example clinical benefits in developmentally disordered populations (Melby-Lervag and Hulme, 2013). Future work in this area that considers the presently suggested relationship between updating specific EF and WM capacity may be productive in informing on the scope of potential effects of WM training and their applicability to atypical child populations.

The present results also pointed toward a role of the cerebellum in updating-specific processes. Cerebellar activation has been linked to performance monitoring during task engagement. Particularly, it has been linked to post-error processing in relation to motor responses (Peterburs et al., 2015). All of the presently included updating tasks incorporated button-press responses, consistent with involvement of post-error motor response processes. Thus, it is possible that the present involvement of cerebellar activity reflects a task specific process, as have been highlighted as important factors to consider in this kind of functional neuroimaging analysis (Chein et al., 2011; Tomasino and Gremese, 2016). Considering such processes, it is interesting to note that a particular role for cross-modal integration of information for WM has been highlighted (Prabhakaran et al., 2000; Zhang et al., 2004; Repovs and Baddeley, 2006). Since the updating tasks involved in the present meta-analysis also involve integration of information across domains, one possibility that warrants further examination is the degree to which updating-specific processes may be inherently task specific.

Notably, our results revealed no updating-specific activation in the child group suggesting a possible distinction between how far updating-specific neural processes can be differentiated in children under 12 years; and those under 18 years. When examining updating subcomponents, age related changes in neural activation linked to retrieval, but not substitution or transformation, have been demonstrated across children, adolescents and young adults (Linares et al., 2016). This is consistent with development in WM capacity throughout childhood and adolescence. Such development follows a linear trajectory with subtle adjustments, in particular, in increased capacity, taking place during adolescence and early adulthood (Gathercole et al., 2004; Satterthwaite et al., 2013). Thus, one interesting possibility highlighted by the present findings is that as WM capacity develops over childhood, so too does the relationship between common and specific components of updating, which allows updating tasks to be performed successfully. A focus for future research may be to assess the development of both dimensions of updating during childhood. And examine if there is a temporal link between improvements in WM capacity and the advancement of the executive component of updating and updating-specific abilities.

Our first-level analysis of switching related activation pointed toward involvement of right parietal-cingulo, left frontal and left occipital (lingual gyrus) regions. These findings must be treated with substantial caution due to the lack of switching data. Yet, they are consistent with previous meta-analyses examining switching-related neural activation in adults (Buchsbaum et al., 2005; Collette et al., 2005; Niendam et al., 2012) and so suggest a general correspondence between children and adults in this respect. Unfortunately due to the low number of switching studies included, a comprehensive examination of switching related activation in children under 12 years was not possible. The present evidence for both a common executive component of switching—which involved left frontal activation—and a switching-specific component, is consistent with previous work in adults (Herd et al., 2014; Reineberg et al., 2015; Smolker et al., 2015) and supports an integrative view of switching in children. However, previous work has pointed toward parietal involvement in a switching-specific process in adults (Collette et al., 2005; Reineberg et al., 2015). But the presently identified switching-specific activity was limited to left occipital regions (lingual gyrus). In interpreting these results, it is again important to consider the limitations of the relatively small amount of data available on switching tasks. However, since all of the presently included switching tasks relied heavily on visual stimuli, the finding is consistent with increased susceptibility to task modality being a feature of less developed cognitive processing (Fisher, 2011; Irving et al., 2011). Interestingly, deficient switching demonstrated in individuals with a particular genetic neurodevelopmental disorder has been associated with greater involvement of occipital; but reduced involvement of frontal parietal brain regions in switching (Woodcock et al., 2010). Thus, an important area for future investigation will be how switching-specific processes change over the course of development. And whether the deficient switching that appears to be evidenced in several neurodevelopmental disorders (Woodcock et al., 2009; Van Eylen et al., 2011), reflects a deficiency in switching-specific processes; the common executive component of switching; or both.

Overall, these findings demonstrate that the neural substrates of executive function in children are part of a superordinate EF network, mainly represented in the fronto-cingulo-parietal cortices. Yet, selective recruitment within these areas and others, such as subcortical regions, is evident when executive process-specific capacity is analyzed. These results are in line with previous meta-analytic research examining EF in adults (Collette et al., 2005; Niendam et al., 2012).

Not dissimilar to other brain imaging meta-analyses, methodological considerations are evident. A limitation of the ALE method is that, with regards to statistical thresholds, inter-study differences are not accounted for- perhaps most notably, the power of each study. Further, this coordinate-based technique does not consider the extent of activation for each cluster but activation location only. Cluster based thresholding does not allow for precise spatial specificity, thus, we must be careful not to make inferences about the statistical significance of a particular location within a given cluster (Woo et al., 2014). Findings should also be regarded as a depiction of positive results, bearing in mind negative results cannot be generated (Cortese et al., 2012).

In addition, the present study did not account for task content (e.g., stimuli type- spatial, letter, number etc.; or response type- motor, verbal). Previous meta-analyses have found EF activation to be task-dependent (Kim et al., 2012). For instance, Simmonds et al. (2008) reported additional “complexity” related activation when they compared simple and complex go/no-go tasks which varied in terms of their working memory demands. Likewise, Swick et al. (2011) acknowledged the need to consider differential processing demands elicited by executive tasks. Upon examination of the neural activation of go/no-go and stop-signal tasks, the authors found concurrent activity for both tasks, whereas non-concurrence appeared in areas of the frontoparietal and cingulo-opercular networks, respectively. It is unfortunate that we were restricted in which tasks we could include in our analysis, as it is possible that the differential processing demands of those tasks had an influence on the patterns of activity identified. Indeed our results may indicate that activation relating to switching-specific and updating-specific abilities reflect processing demands necessary for respective task completion. Yet, since our analyses did not rely on only one particular task, the task-specific influence on our results was minimized. Nonetheless, in order to demonstrate a more complete neural picture of EF performance, future meta-analytic study should assess neural activity associated with EF task-specific components, which may in turn help to promote more effective EF measurement.

A further limitation of the present study is the broad age range used in the dataset. In addition to this, as some papers included in the analysis did not report detailed age demographics (see Table 2), there may be variability in the overall age range reported. Moreover, a clear limitation is the lack of switching studies that were available for inclusion. Thus, the present results relating to switching, particularly in the higher-level comparisons with other executive processes, should be treated with caution. While there has been considerable interest in examining the neural correlates of switching using fMRI, most of these studies do not include data from typical children and/or have not examined the contrasts appropriate for isolating the presently studied construct of switching. This may be because switching has been examined at a more sub-componential level e.g., the focus of the literature does not seem to be in examining switching *per-se* but instead how it works. Perhaps if a model of EF can be applied to children, which includes switching as a basic construct, this might facilitate more future attention on the construct of switching itself.

Finally, it is important to acknowledge the assumption made in the present analyses, based on our theoretical position. That is, isolating common executive activity based on tests tapping only two putative executive processes (Figure 1B), served an equivalent role to isolating such activity based on tests tapping three or more executive processes (Figure 1A). We were able to test this assumption on a small scale in our control analyses of updating, which pointed toward consistency with our primary analyses. We also conducted further second-level analyses which examined the shared and non-shared activation between maps of common executive, which included all tasks pertaining to inhibition, switching and updating and one of the executive processes. These analyses assessed whether inclusion of this data would bias the patterns of overlap and distinction. As expected, results showed shared overlap when each executive process was compared to the “inclusive” common executive map (with more significant clusters identified than in the primary analyses reported here). But no distinct clusters in contrast analyses were found in any of the analyses (Supplementary Materials Sections I–K). Thus, supporting the existence of a bias toward identification of conjunctive activation if any of the same studies are included in two maps compared in second-level analyses. These findings support our assumption. Nevertheless, the nature of the limitation itself meant that it could not be tested directly. For example, second-level comparison of a common executive map comprising inhibition, switching and updating studies; to one comprising only the inhibition and switching studies; would be biased toward identification of conjunctive activation.

In conclusion, the findings suggest that a structural model of EF—proposing one common underlying, and multiple separable processes—can be applied during development. However, in line with recent behavioral evidence, it does not appear that inhibition can be distinguished from the common process. And, updating and switching appear separable when considering adolescents alongside children. But, in children, these processes may not be separable. Thus, due to the complex nature of development and the changing structural climate of EF throughout childhood (Tsujimoto et al., 2007; Shing et al., 2010; Brydges et al., 2014; Lerner and Lonigan, 2014; Howard et al., 2015), perhaps a new systematic developmental model is needed. The model should encourage careful measurement of common and process-specific components. Previous meta-analytic study has reported effects of task modality on EF performance in children (Booth et al., 2010). However, the influence of non-executive factors on EF performance at a neural level has not yet been investigated. As a result, future examination is warranted, which could inform on valid EF measurement. Only then, can we begin to systematically amalgamate knowledge acquired through understanding the neural infrastructure of EF in development, to behavior—in particular, executive dysfunction in clinical populations.

## Author contributions

All authors made substantial contributions to research design, drafting and final approval of the manuscript. RM conducted the literature searches and analyses as a part of her doctoral research. KW acted as RM's principal supervisor and TR acted as RM's second supervisor.

### Conflict of interest statement

The authors declare that the research was conducted in the absence of any commercial or financial relationships that could be construed as a potential conflict of interest.
