# Supplementary material for: Informing the Structure of Executive Function in Children: A Meta-Analysis of Functional Neuroimaging Data
Source: Front Hum Neurosci. 2017 Apr 7;11:154. doi: 10.3389/fnhum.2017.00154 (PMC5383671; doi:10.3389/fnhum.2017.00154)
Supplement: Supplementary file 1 [file DataSheet1.DOCX]

**Supplementary Material**

**A. Detailed cluster demographics for first-level analyses for Common Executive, Inhibition, Updating & Switching in the child/adolescent group**

|  | Cluster # | Volume (mm^3) | Weighted Centre (x,y,z) | | | Region |
| --- | --- | --- | --- | --- | --- | --- |
| Common Executive | 1 | 8648 | 1.01 | 15.75 | 46.18 | Left Medial Frontal Gyrus (BA 32 & 6) |
|  | 2 | 5312 | 29.77 | -55.81 | 48.58 | Right Inferior Parietal Lobule (BA 40) |
|  |  |  |  |  |  | Right Superior Parietal Lobule (BA 7) |
|  |  |  |  |  |  | Right Precuneus (BA 7) |
|  | 3 | 4880 | 39.49 | 21.29 | -4.9 | Right Insula |
|  |  |  |  |  |  | Right Claustrum |
|  | 4 | 2376 | -30.83 | -49.47 | 48.18 | Left Inferior Parietal Lobule (BA 40) |
|  |  |  |  |  |  | Left Superior Parietal Lobule (BA 7) |
|  |  |  |  |  |  | Left Precuneus (BA 7) |
|  | 5 | 1760 | -32.59 | 20.39 | 1.76 | Left Insula (BA 13) |
|  | 6 | 1496 | 36.12 | 42.31 | 31.02 | Right Middle Frontal Gyrus (BA 9) |
|  | 7 | 1368 | -46.65 | 6.2 | 31.79 | Left Precentral Gyrus (BA 6) |
|  |  |  |  |  |  | Left Middle Frontal Gyrus (BA 9) |
|  | 8 | 1176 | -22.26 | 6.12 | 53.7 | Left Frontal Sub-Gyral Matter (BA 6) |
|  | 9 | 904 | 47.19 | 5.81 | 31.56 | Right Precentral Gyrus (BA 6) |
|  | 10 | 840 | 43.89 | -61.17 | -8.59 | Right Fusiform Gyrus (BA 37 & 19) |
|  | 11 | 664 | 30.34 | 9.71 | 56.72 | Right Frontal Sub-Gyral Matter (BA 6) |
|  | 12 | 584 | -23.23 | -65.86 | 39.7 | Left Precuneus (BA 7) |
|  | 13 | 520 | -43.79 | 31.16 | 32.59 | Left Middle Frontal Gyrus (BA 9) |
|  | 14 | 448 | 36.23 | -57.1 | -26.51 | Right Culmen |
|  | 15 | 448 | 26.91 | -0.14 | 48.53 | Right Middle Frontal Gyrus (BA 6) |
|  | 16 | 440 | -7.95 | -67.3 | 60.06 | Left Superior Parietal Lobule (BA 7) |
|  |  |  |  |  |  | Left Precuneus (BA 7) |
|  | 17 | 432 | 10.78 | 17.18 | -2.69 | Head of the Right Caudate nucleus |
|  | 18 | 384 | 47.24 | -20.98 | 44.7 | Right Postcentral Gyrus (BA 2) |
|  | 19 | 360 | -40.63 | -61.03 | -26.43 | Left Culmen |
|  |  |  |  |  |  | Left Posterior Lobe of Cerebellum |
|  | 20 | 360 | -11.79 | 1.57 | 14.52 | Body of the Left Caudate nucleus |
|  | 21 | 304 | 55.16 | -43.06 | 51.89 | Right Inferior Parietal Lobule (BA 40) |
|  | 22 | 248 | 23.74 | -68.91 | 33.17 | Right Precuneus (BA 7) |
|  | 23 | 200 | -53.11 | -4.18 | 44.06 | Left Precentral Gyrus (BA 4) |
|  | 24 | 184 | 4.49 | -7.21 | 43.72 | Right Cingulate Gyrus (BA 24) |
|  | 25 | 144 | 55.11 | -42.99 | 31.98 | Right Inferior Parietal Lobule (BA 40) |
|  | 26 | 120 | 5.47 | -17.07 | -9.21 | Red Nucleus, Right Midbrain |
|  | 27 | 120 | -39.86 | -79.71 | -3.06 | Left Inferior Occipital Gyrus (BA 19) |
|  | 28 | 112 | -27.59 | -78.14 | 23.28 | Left Middle Occipital Gyrus (BA 19) |
|  | 29 | 104 | -44.95 | 26.77 | 1.99 | Left Inferior Frontal Gyrus (BA 13) |
| Inhibition | 1 | 6520 | 1.92 | 13.99 | 46.49 | Right Cingulate Gyrus (BA 32) |
|  |  |  |  |  |  | Left Medial Frontal Gyrus (BA 32 & 6) |
|  |  |  |  |  |  | Right Medial Frontal Gyrus (BA 6) |
|  |  |  |  |  |  | Right Superior Frontal Gyrus (BA 6) |
|  | 2 | 4432 | 43.01 | 20.29 | -5.22 | Right Extra-Nuclear. (BA 47) |
|  |  |  |  |  |  | Right Insula (BA 13) |
|  | 3 | 2560 | 27.09 | -58.59 | 51.05 | Right Precuneus (BA 7) |
|  |  |  |  |  |  | Right Inferior Parietal Lobule (BA 40) |
|  |  |  |  |  |  | Right Superior Parietal Lobule (BA 7) |
|  | 4 | 1776 | -35.74 | 20.86 | 2.41 | Left Insula (BA 13) |
|  |  |  |  |  |  | Left Inferior Frontal Gyrus (BA 13) |
|  |  |  |  |  |  | Left Inferior Frontal Gyrus (BA 45) |
|  | 5 | 952 | 10.97 | 17.27 | -2.71 | Head of the Right Caudate nucleus |
|  | 6 | 680 | 35.38 | 42.92 | 33.16 | Right Middle Frontal Gyrus (BA 9) |
|  | 7 | 640 | 43.47 | -58.79 | -9.03 | Right Fusiform Gyrus (BA 37) |
|  | 8 | 456 | 55.38 | -43.71 | 32.24 | Right Inferior Parietal Lobule (BA 40) |
|  | 9 | 408 | -39.28 | -79.32 | -3.17 | Left Inferior Occipital Gyrus (BA 19) |
|  | 10 | 400 | -35.7 | 41.04 | 24.13 | Left Superior Frontal Gyrus (BA 9) |
|  | 11 | 376 | 59.85 | -40.89 | 13.09 | Right Superior Temporal Gyrus (BA 22) |
|  | 12 | 336 | -10.28 | 5.54 | 12.46 | Body of the Left Caudate nucleus |
|  | 13 | 336 | 26.75 | 0 | 47.24 | Right Middle Frontal Gyrus (BA 6) |
|  | 14 | 320 | -24.37 | -55.79 | 59.83 | Left Precuneus (BA 7) |
|  | 15 | 272 | 22.44 | -70.53 | 34 | Right Precuneus (BA 31) |
|  | 16 | 256 | -50.54 | 8.07 | -3.84 | Left Superior Temporal Gyrus (BA 22) |
|  | 17 | 232 | 50.01 | 5.99 | 30.01 | Right Inferior Frontal Gyrus (BA 6) |
|  | 18 | 216 | 34.02 | -57.69 | -24.33 | Right Culmen |
|  | 19 | 168 | 11.74 | 1.55 | 68.12 | Right Superior Frontal Gyrus (BA 6) |
|  | 20 | 160 | -29.09 | -51.5 | 49.08 | Left Precuneus (BA 7) |
|  |  |  |  |  |  | Left Superior Parietal Lobule (BA 7) |
| Updating | 1 | 3856 | -0.36 | 17.41 | 46.32 | Left Medial Frontal Gyrus (BA 6) |
|  |  |  |  |  |  | Left Cingulate Gyrus (BA 24) |
|  |  |  |  |  |  | Left Superior Frontal Gyrus (BA 6) |
|  | 2 | 1640 | 49.33 | 15.76 | 21.81 | Right Inferior Frontal Gyrus (BA 44 & 9) |
|  |  |  |  |  |  | Right Precentral Gyrus (BA 9) |
|  |  |  |  |  |  | Right Middle Frontal Gyrus (BA 9) |
|  | 3 | 1504 | 40.12 | -45.88 | 44.96 | Right Inferior Parietal Lobule (BA 40) |
|  | 4 | 1232 | -40.7 | -66.06 | -30.16 | Left Posterior Lobe of Cerebellum |
|  |  |  |  |  |  | Left Posterior Lobe of Cerebellum |
|  | 5 | 1192 | 35.24 | 22.12 | -2.56 | Right Insula |
|  | 6 | 1176 | 30.29 | 9.54 | 56.77 | Right Frontal Sub-Gyral Matter (BA 6) |
|  | 7 | 1040 | -24.69 | 7.46 | 52.41 | Left Frontal Sub-Gyral Matter (BA 6) |
|  | 8 | 1016 | -33.45 | -45.37 | 42.4 | Left Inferior Parietal Lobule (BA 40) |
|  | 9 | 880 | 31.48 | -62.67 | 37.92 | Right Precuneus (BA 7) |
|  | 10 | 680 | -32.05 | 19.94 | 0.6 | Left Claustrum |
|  | 11 | 656 | -8.54 | -65.5 | 61.93 | Left Superior Parietal Lobule (BA 7) |
|  | 12 | 520 | -40.99 | 1.94 | 35.51 | Left Precentral Gyrus (BA 6) |
|  |  |  |  |  |  | Left Inferior Frontal Gyrus (BA 6) |
|  | 13 | 488 | -20.99 | -63.99 | 41.96 | Left Precuneus (BA 7) |
|  | 14 | 384 | 38.68 | -60.09 | -34.57 | Right Anterior Lobe of Cerebellum |
|  |  |  |  |  |  | Right Posterior Lobe of Cerebellum |
|  | 15 | 360 | 53.85 | -42.37 | 52.63 | Right Inferior Parietal Lobule (BA 40) |
|  | 16 | 320 | 37.46 | 35.67 | 26.99 | Right Middle Frontal Gyrus (BA 9) |
|  | 17 | 288 | -31.71 | -51 | 56.59 | Left Superior Parietal Lobule (BA 7) |
|  | 18 | 280 | -43.21 | -5.9 | 55.21 | Left Precentral Gyrus (BA 4) |
|  | 19 | 264 | 16.82 | -68.28 | 46.47 | Right Precuneus (BA 7) |
|  | 20 | 224 | -14.07 | -2.08 | 17.21 | Body of the Left Caudate nucleus |
|  | 21 | 192 | 37.35 | -2.5 | 52.44 | Right Precentral Gyrus (BA 6) |
|  | 22 | 152 | -38.55 | 25.92 | 26.42 | Left Middle Frontal Gyrus (BA 9) |
|  | 23 | 128 | -54.39 | 24.37 | 34.38 | Left Middle Frontal Gyrus (BA 9) |
|  | 24 | 112 | 17.58 | -74.59 | 49.71 | Right Precuneus (BA 7) |
|  | 25 | 104 | 52.17 | 0.94 | 43.81 | Right Precentral Gyrus (BA 6) |
| Switching | 1 | 488 | 48.52 | -21.47 | 44 | Right Postcentral Gyrus (BA 2) |
|  | 2 | 288 | 4.23 | -8.34 | 44.05 | Right Cingulate Gyrus (BA 24) |
|  | 3 | 272 | -6.8 | -72.46 | 4.07 | Left Lingual Gyrus (BA 18) |
|  | 4 | 168 | -46.69 | 3.31 | 29.07 | Left Precentral Gyrus (BA 6) |

BA, Brodmann area.

**B. Detailed cluster demographics for first-level analyses for Common Executive and Inhibition in the child group**

|  | Cluster # | Volume (mm^3) | Weighted Centre (x,y,z) | | | Region |
| --- | --- | --- | --- | --- | --- | --- |
| Common Executive | 1 | 7352 | 0.38 | 15.48 | 46.66 | Left Medial Frontal Gyrus (BA 32 & 6) |
|  | 2 | 2024 | 39.14 | -46.52 | 44.61 | Right Inferior Parietal Lobule (BA 40) |
|  | 3 | 1704 | 34.63 | 21.08 | 2.19 | Right Claustrum |
|  |  |  |  |  |  | Right Insula |
|  | 4 | 1504 | 22.32 | -63.49 | 46.28 | Right Precuneus (BA 7) |
|  | 5 | 1120 | -19.66 | 4.08 | 55.94 | Left Frontal Sub-Gyral Matter (BA 6) |
|  | 6 | 1000 | 28.48 | -0.57 | 48.92 | Right Middle Frontal Gyrus (BA 6) |
|  |  |  |  |  |  | Right Precentral Gyrus (BA 6) |
|  | 7 | 840 | 35.99 | 42.97 | 32.26 | Right Middle Frontal Gyrus (BA 9) |
|  | 8 | 696 | 53.5 | 10.48 | 16.61 | Right Inferior Frontal Gyrus (BA 44 & 9) |
|  | 9 | 680 | -31.78 | 21.67 | 2.75 | Left Insula (BA 13) |
|  | 10 | 456 | -10.39 | 4.71 | 12.5 | Body of the Left Caudate nucleus |
|  | 11 | 400 | 16.53 | -77.48 | 50.22 | Right Precuneus (BA 19) |
|  | 12 | 320 | 49.83 | 17.55 | -11.37 | Right Inferior Frontal Gyrus (BA 47) |
|  | 13 | 296 | -40.08 | 1.59 | 36.91 | Left Precentral Gyrus (BA 6) |
|  | 14 | 264 | 54.8 | -41.78 | 31.05 | Right Inferior Parietal Lobule (BA 40) |
|  | 15 | 256 | 54.17 | -42.28 | 52.3 | Right Inferior Parietal Lobule (BA 40) |
|  | 16 | 256 | -43.53 | -6 | 54.58 | Left Precentral Gyrus (BA 4) |
|  | 17 | 248 | 43.61 | -58.2 | -10.06 | Right Fusiform Gyrus (BA 37) |
|  | 18 | 240 | 42.2 | -0.47 | 37.45 | Right Precentral Gyrus (BA 6) |
|  | 19 | 232 | 24.15 | 45.28 | -11.59 | Right Medial Frontal Gyrus (BA 10) |
|  | 20 | 224 | -22.66 | 19.07 | 54.58 | Left Superior Frontal Gyrus (BA 6) |
|  | 21 | 216 | -20.53 | -64.49 | 39.94 | Left Precuneus (BA 7) |
|  | 22 | 208 | 44.37 | 22.46 | 37.25 | Right Middle Frontal Gyrus (BA 8) |
|  |  |  |  |  |  | Right Precentral Gyrus (BA 9) |
|  | 23 | 208 | -6.63 | -71.86 | 55.37 | Left Precuneus (BA 7) |
|  | 24 | 192 | -34.34 | -51.51 | 45.43 | Left Inferior Parietal Lobule (BA 40) |
|  | 25 | 184 | 15.92 | 18.71 | -2.88 | Head of the Right Caudate nucleus |
|  | 26 | 160 | 29.89 | 10.01 | 57.91 | Right Frontal Sub-Gyral Matter (BA 6) |
|  | 27 | 152 | -15.6 | -98.73 | 6.72 | Left Cuneus (BA 17) |
|  | 28 | 144 | -0.44 | 3.54 | 22.22 | Left Cingulate Gyrus (BA 24) |
|  | 29 | 120 | 28.41 | 59.46 | 10.94 | Right Middle Frontal Gyrus (BA 10) |
|  | 30 | 120 | 24.28 | -62.01 | 63.32 | Right Superior Parietal Lobule (BA 7) |
| Inhibition | 1 | 4288 | 0.88 | 15.86 | 46.01 | Left Medial Frontal Gyrus (BA 32) |
|  |  |  |  |  |  | Left Superior Frontal Gyrus (BA 6) |
|  |  |  |  |  |  | Right Medial Frontal Gyrus (BA 8 & 6) |
|  |  |  |  |  |  | Right Superior Frontal Gyrus (BA 6) |
|  | 2 | 904 | 35.45 | 43.43 | 33.05 | Right Middle Frontal Gyrus (BA 9) |
|  | 3 | 584 | -10.03 | 5.2 | 12.61 | Body of the Left Caudate nucleus |
|  | 4 | 472 | 15.4 | 18.59 | -2.8 | Head of the Right Caudate nucleus |
|  | 5 | 440 | 26.86 | -0.21 | 47.1 | Right Middle Frontal Gyrus (BA 6) |
|  | 6 | 408 | 34.13 | 20.97 | 7.16 | Right Insula (BA 13) |
|  | 7 | 400 | 55.07 | -41.85 | 31.08 | Right Inferior Parietal Lobule (BA 40) |
|  | 8 | 384 | 43.34 | -58.48 | -10.12 | Right Fusiform Gyrus (BA 37) |
|  | 9 | 384 | 34.71 | -50.5 | 45.13 | Right Superior Parietal Lobule (BA 7) |
|  | 10 | 312 | 26.91 | -63.06 | 47.23 | Right Superior Parietal Lobule (BA 7) |
|  | 11 | 280 | 51.68 | 16.78 | -10.6 | Right Inferior Frontal Gyrus (BA 47) |
|  | 12 | 256 | -22.11 | 19.7 | 55.47 | Left Superior Frontal Gyrus (BA 6) |
|  | 13 | 200 | -45.92 | 7.43 | -1.61 | Left Insula (BA 13) |
|  | 14 | 152 | -36.46 | -77.23 | -5.13 | Left Inferior Occipital Gyrus (BA 19) |
|  | 15 | 128 | -16.14 | 2.87 | 60.49 | Left Middle Frontal Gyrus (BA 6) |
|  | 16 | 120 | -11.34 | 16.54 | -1.34 | Head of the Left Caudate nucleus |
|  | 17 | 120 | 51.19 | 15.32 | 2.27 | Right Precentral Gyrus (BA 44) |
|  | 18 | 112 | 39.86 | -40.41 | 44 | Right Inferior Parietal Lobule (BA 40) |

BA, Brodmann area.

**C. Second-level Conjunction and Contrast Analyses for Common Executive (update, switch) and Inhibition in the child/adolescent group**

|  | Cluster # | Volume (mm^3) | Weighted Center (x,y,z) | | | Region |
| --- | --- | --- | --- | --- | --- | --- |
| Conjunction | 1 | 2776 | 0.66 | 16.22 | 45.66 | Left Medial Frontal Gyrus (BA 32) |
|  |  |  |  |  |  | Left Superior Frontal Gyrus (BA 6) |
|  | 2 | 432 | -32.07 | 20.87 | 1.35 | Left Insula (BA 13) |
|  | 3 | 320 | 37.35 | 22.66 | -5.77 | Right Insula |
|  | 4 | 96 | 38.99 | -49.98 | 46.99 | Right Inferior Parietal Lobule (BA 40) |
|  | 5 | 56 | 32.3 | 20.3 | 4.54 | Right Claustrum |
|  | 6 | 48 | 29.31 | -61.68 | 46.67 | Right Superior Parietal Lobule (BA 7) |
|  | 7 | 8 | 46 | 6 | 30 | Right Precentral Gyrus (BA 6) |
|  | 8 | 8 | 26 | -62 | 44 | Right Precuneus (BA 7) |
|  | 9 | 8 | -32 | -52 | 54 | Left Superior Parietal Lobule (BA 7) |
|  | 10 | 8 | -32 | -54 | 56 | Left Superior Parietal Lobule (BA 7) |
| Difference | No clusters found | |  |  |  |  |

BA, Brodmann area.

*[Image 1]*

**D. Second-level Conjunction and Contrast Analyses for Common Executive (update, switch) and Inhibition in the child group**

|  | Cluster # | Volume (mm^3) | Weighted Center (x,y,z) | | | Region |  |  |
| --- | --- | --- | --- | --- | --- | --- | --- | --- |
| Conjunction | 1 | 2160 | 0.2 | 16.1 | 45.8 | Left Medial Frontal Gyrus (BA 32) | | |
|  |  |  |  |  |  | Left Medial Frontal Gyrus (BA 6) | | |
|  |  |  |  |  |  | Right Cingulate Gyrus (BA 32) | | |
|  |  |  |  |  |  | Right Medial Frontal Gyrus (BA 6) | | |
|  | 2 | 96 | 32.3 | 20.5 | 5.3 | Right Claustrum | | |
|  | 3 | 48 | 40.7 | -41 | 43.4 | Right Inferior Parietal Lobule (BA 40) | | |
|  | 4 | 48 | 27 | -62.7 | 44.7 | Right Precuneus (BA 7) | | |
|  | 5 | 40 | 38 | -49.2 | 45.6 | Right Inferior Parietal Lobule (BA 40) | | |
| Difference | No clusters found | |  |  |  |  | | |

BA, Brodmann area.

*[Image 2]*

**E. Second-level Conjunction and Contrast Analyses for Common Executive (inhibit, switch) and Updating in the child/adolescent group**

|  | Cluster # | Volume (mm^3) | Weighted Centre (x,y,z) | | | Region |
| --- | --- | --- | --- | --- | --- | --- |
| Conjunction | 1 | 2576 | 0.72 | 16.18 | 46.52 | Left Medial Frontal Gyrus (BA 6 & 32) |
|  |  |  |  |  |  | Left Superior Frontal Gyrus (BA 6) |
|  | 2 | 440 | -32.01 | 21.03 | 1.57 | Left Insula (BA 13) |
|  | 3 | 280 | 37.46 | 23.09 | -6.05 | Right Insula |
|  | 4 | 120 | -30.07 | -47.71 | 42.8 | No Grey Matter found |
|  | 5 | 120 | 38.34 | -49.99 | 46.69 | Right Inferior Parietal Lobule (BA 40) |
|  | 6 | 72 | 28.02 | -61.99 | 46.65 | Right Superior Parietal Lobule (BA 7) |
|  | 7 | 56 | 32.56 | 20.32 | 4.55 | Right Claustrum |
|  | 8 | 40 | -45.2 | 4.81 | 32 | Left Inferior Frontal Gyrus (BA 6) |
|  |  |  |  |  |  | Left Precentral Gyrus (BA 6) |
| Difference | 1 | 1136 | 30.27 | 9.18 | 56.7 | Right Frontal Sub-Gyral Matter (BA 6) |
|  |  |  |  |  |  | Right Middle Frontal Gyrus (BA 6) |
|  | 2 | 760 | 45.34 | 19.75 | 23.99 | Right Middle Frontal Gyrus (BA 9) |
|  |  |  |  |  |  | Right Precentral Gyrus (BA 9) |
|  | 3 | 672 | -40.93 | -67.21 | -31.57 | Left Posterior Lobe of Cerebellum |
|  |  |  |  |  |  | Left Posterior Lobe of Cerebellum |
|  | 4 | 144 | 38.79 | -63.16 | -39.27 | Right Posterior Lobe of Cerebellum |

BA, Brodmann area.

**F. Second-level Conjunction and Contrast Analyses for Common Executive (inhibit, switch) and Updating in the child group**

|  | Cluster # | Volume (mm^3) | Weighted Center (x,y,z) | | | Region |  |  |
| --- | --- | --- | --- | --- | --- | --- | --- | --- |
| Conjunction | 1 | 2208 | 0.3 | 16.2 | 45.8 | Left Medial Frontal Gyrus (BA 32) | | |
|  |  |  |  |  |  | Left Medial Frontal Gyrus (BA 6) | | |
|  |  |  |  |  |  | Right Cingulate Gyrus (BA 32) | | |
|  |  |  |  |  |  | Right Medial Frontal Gyrus (BA 6) | | |
|  | 2 | 104 | 32.6 | 20.6 | 5.2 | Right Claustrum | | |
|  | 3 | 56 | 40.6 | -41.1 | 43.7 | Right Inferior Parietal Lobule (BA 40) | | |
|  | 4 | 48 | 27 | -62.7 | 44.7 | Right Precuneus (BA 7) | | |
|  | 5 | 40 | 38 | -49.2 | 45.6 | Right Inferior Parietal Lobule (BA 40) | | |
|  | 6 | 8 | 36 | -48 | 42 | Right Inferior Parietal Lobule (BA 40) | | |
| Difference | No clusters found | |  |  |  |  | | |

BA, Brodmann area.

*[Image 3]*

**G. Second-level Conjunction and Contrast Analyses for Common Executive (inhibit, update) and Switching**

|  | Cluster # | Volume (mm^3) | Weighted Centre (x,y,z) | | | Region |
| --- | --- | --- | --- | --- | --- | --- |
| Conjunction | 1 | 88 | -45.28 | 3.59 | 30.14 | Left Precentral Gyrus (BA 6) |
| Difference | 1 | 192 | -5.6 | -72.66 | 3.18 | Left Lingual Gyrus (BA 18) |

BA, Brodmann area.

**H. Contrast clusters from the Control Analyses for Common Executive and Updating**

| Cluster # | Volume (mm^3) | Weighted Centre (x,y,z) | | | Region |
| --- | --- | --- | --- | --- | --- |
| 1 | 216 | 52.37 | -42.44 | 55.78 | Right Inferior Parietal Lobule (BA 40) |
| 2 | 304 | 37.81 | -1.79 | 53.17 | Right Middle Frontal Gyrus (BA 6 ) |
| 3 | 104 | -30.88 | -69.72 | -25.72 | Left Posterior Lobe of Cerebellum |

BA, Brodmann area.

*[Image 4]*

**I. Second-level Conjunction and Contrast Analyses for Common Executive (inclusive) and Inhibition**

|  | Cluster # | Volume (mm^3) | Weighted Centre (x,y,z) | | | Region |
| --- | --- | --- | --- | --- | --- | --- |
| Conjunction | 1 | 5976 | 1.91 | 14.43 | 46.17 | Right Cingulate Gyrus (BA 32) |
|  |  |  |  |  |  | Left Medial Frontal Gyrus (BA 32 & 6) |
|  |  |  |  |  |  | Right Medial Frontal Gyrus (BA 6) |
|  |  |  |  |  |  | Right Superior Frontal Gyrus (BA 6) |
|  | 2 | 3464 | 42 | 20.82 | -6.04 | Right Extra-Nuclear (BA 47) |
|  |  |  |  |  |  | Right Insula (BA 13) |
|  | 3 | 1616 | 23.27 | -61.55 | 52.44 | Right Precuneus (BA 7) |
|  |  |  |  |  |  | Right Superior Parietal Lobule (BA 7) |
|  | 4 | 1232 | -32.96 | 20.74 | 2.61 | Left Insula (BA 13) |
|  | 5 | 744 | 35.75 | -52.09 | 46.77 | Right Inferior Parietal Lobule (BA 40) |
|  | 6 | 544 | 35.77 | 42.99 | 32.97 | Right Middle Frontal Gyrus (BA 9) |
|  | 7 | 512 | 43.61 | -59.02 | -8.75 | Right Fusiform Gyrus (BA 37) |
|  | 8 | 432 | 10.78 | 17.19 | -2.69 | Head of the Right Caudate nucleus |
|  | 9 | 288 | 26.76 | -0.14 | 47.56 | Right Middle Frontal Gyrus (BA 6) |
|  | 10 | 232 | -24.83 | -55.66 | 59.6 | Left Precuneus (BA 7) |
|  | 11 | 224 | 49.95 | 6.05 | 30.07 | Right Inferior Frontal Gyrus (BA 6) |
|  | 12 | 176 | 34.34 | -57.48 | -24.55 | Right Culmen |
|  | 13 | 168 | 23.17 | -69.83 | 33.83 | Right Precuneus (BA 31) |
|  | 14 | 160 | -10.13 | 4.67 | 12.79 | Body of the Left Caudate nucleus |
|  | 15 | 160 | -29.09 | -51.5 | 49.08 | Left Precuneus (BA 7) |
|  |  |  |  |  |  | Left Superior Parietal Lobule (BA 7) |
|  | 16 | 144 | 55.11 | -43 | 31.97 | Right Inferior Parietal Lobule (BA 40) |
|  | 17 | 120 | -39.86 | -79.71 | -3.06 | Left Inferior Occipital Gyrus (BA 19) |
|  | 18 | 104 | -44.94 | 26.76 | 1.97 | Left Inferior Frontal Gyrus (BA 13) |
| Difference | No Clusters found | |  |  |  |  |

BA, Brodmann area.

**J. Second-level Conjunction and Contrast Analyses for Common Executive (inclusive) and Updating**

|  | Cluster # | Volume (mm^3) | Weighted Center (x,y,z) | | | Region |
| --- | --- | --- | --- | --- | --- | --- |
| Conjunction | 1 | 3840 | -0.34 | 17.42 | 46.31 | Left Medial Frontal Gyrus (BA 6) |
|  |  |  |  |  |  | Left Cingulate Gyrus (BA 24) |
|  |  |  |  |  |  | Left Superior Frontal Gyrus (BA 6) |
|  | 2 | 1272 | 39.96 | -45.89 | 44.99 | Right Inferior Parietal Lobule (BA 40) |
|  |  |  |  |  |  | Right Inferior Parietal Lobule (BA 40) |
|  | 3 | 1192 | 35.24 | 22.12 | -2.56 | Right Insula |
|  | 4 | 808 | -33.08 | -45.73 | 42.43 | Left Inferior Parietal Lobule (BA 40) |
|  | 5 | 808 | -24.17 | 7.34 | 52.62 | Left Frontal Sub-Gyral (BA 6) |
|  | 6 | 680 | -32.05 | 19.94 | 0.6 | Left Claustrum |
|  | 7 | 664 | 30.35 | 9.68 | 56.72 | Right Frontal Sub-Gyral Matter (BA 6) |
|  | 8 | 360 | 30.24 | -61.8 | 45.49 | Right Precuneus (BA 7) |
|  | 9 | 320 | -8.27 | -66.06 | 61.66 | Left Superior Parietal Lobule (BA 7) |
|  | 10 | 296 | -21.43 | -64.71 | 40.62 | Left Precuneus (BA 7) |
|  | 11 | 288 | -31.71 | -51 | 56.59 | Left Superior Parietal Lobule (BA 7) |
|  | 12 | 240 | 37.6 | 35.8 | 27.58 | Right Middle Frontal Gyrus (BA 9) |
|  | 13 | 232 | 54.57 | -42.49 | 52.19 | Right Inferior Parietal Lobule (BA 40) |
|  | 14 | 216 | -42.74 | 3.12 | 33.76 | Left Precentral Gyrus (BA 6) |
|  |  |  |  |  |  | Left Inferior Frontal Gyrus (BA 6) |
|  | 15 | 160 | -42.47 | -65.46 | -27.58 | Left Posterior Lobe of Cerebellum |
|  | 16 | 120 | -14 | -1.53 | 16.84 | Left Caudate |
|  | 17 | 112 | 15.97 | -66.32 | 47.97 | Right Precuneus (BA 7) |
|  | 18 | 104 | 38.34 | -57.22 | -29.75 | Right Anterior Lobe of Cerebellum |
|  | 19 | 32 | -38.01 | 28 | 27.48 | Left Middle Frontal Gyrus (BA 9) |
|  | 20 | 8 | -40 | -62 | -24 | Left Posterior Lobe of Cerebellum |
| Difference | No clusters found | |  |  |  |  |

BA, Brodmann area.

**K. Second-level Conjunction and Contrast Analyses for Common Executive (inclusive) and Switching**

|  | Cluster # | Volume (mm^3) | Weighted Center (x,y,z) | | | Region |
| --- | --- | --- | --- | --- | --- | --- |
| Conjunction | 1 | 320 | 47.9 | -21.33 | 44.23 | Right Postcentral Gyrus (BA 2) |
|  | 2 | 160 | 4.45 | -7.65 | 43.89 | Right Cingulate Gyrus (BA 24) |
|  | 3 | 152 | -46.17 | 3.24 | 29.47 | Left Precentral Gyrus (BA 6) |
| Difference | No clusters found | |  |  |  |  |

BA, Brodmann area.

**Image 1. Second-level Conjunction Analysis for Common Executive (update, switch) and Inhibition in the child/adolescent group (x=5, y=19, z=47).** ALE maps showing the significant conjunction clusters of Common Executive (update, switch) and Inhibition in the child/adolescent group (10 clusters). No contrast clusters were found.

**Image 2. Second-level Conjunction Analysis for Common Executive (update, switch) and Inhibition in the child group (x=5, y=-40, z=44).** ALE maps showing the significant conjunction clusters of Common Executive (update, switch) and Inhibition in the child group (5 clusters). No contrast clusters were found.

**Image 3.** **Second-level Conjunction Analysis for Common Executive (inhibit, switch) and Updating in the child group (x=3, y=-42, z=44).** Significant conjunction analysis results for common executive (inhibit, switch) and updating in the child group (6 clusters). No contrast clusters were found.

**Image 4. Contrast clusters from the Control Analyses for Common Executive and Updating (x=-30, y=1, z=55).** Significant contrast clusters from 2 control analyses for Common Executive and Updating (2 clusters resulting from one analysis (red) and 1 cluster (green) from another analysis) are displayed.
