# Supplementary figures and images for: Informing the Structure of Executive Function in Children: A Meta-Analysis of Functional Neuroimaging Data
Source: Front Hum Neurosci. 2017 Apr 7;11:154. doi: 10.3389/fnhum.2017.00154 (PMC5383671; doi:10.3389/fnhum.2017.00154)

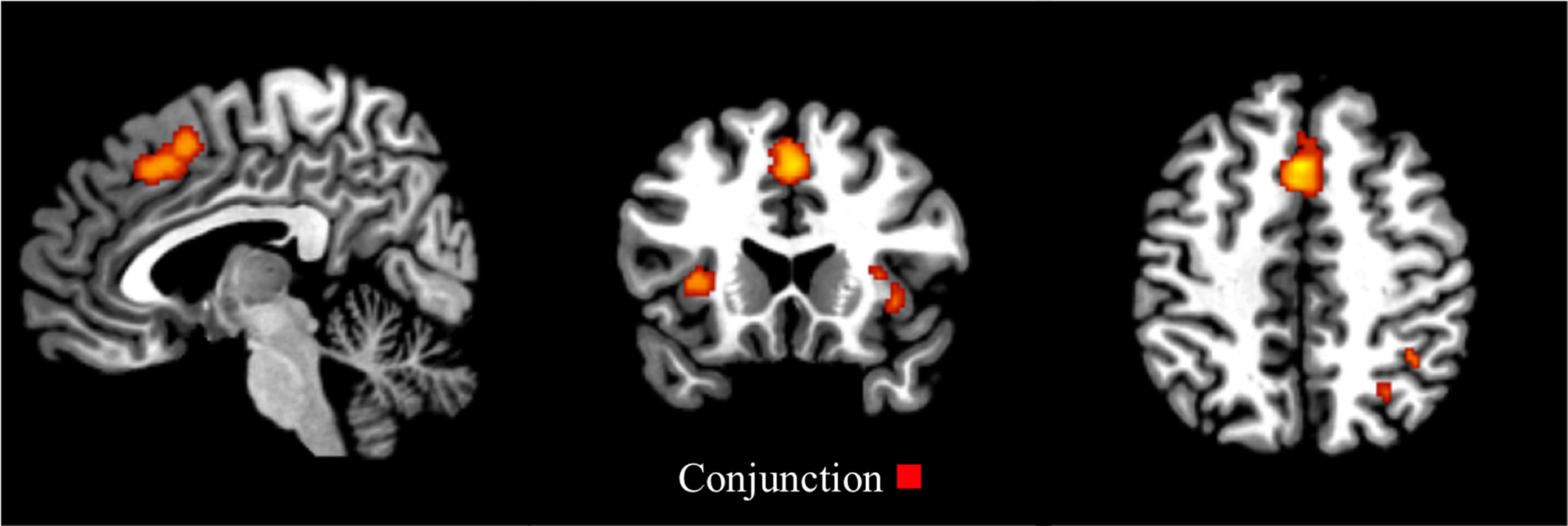

Supplement: Supplementary file 2 [file Image1.TIFF]

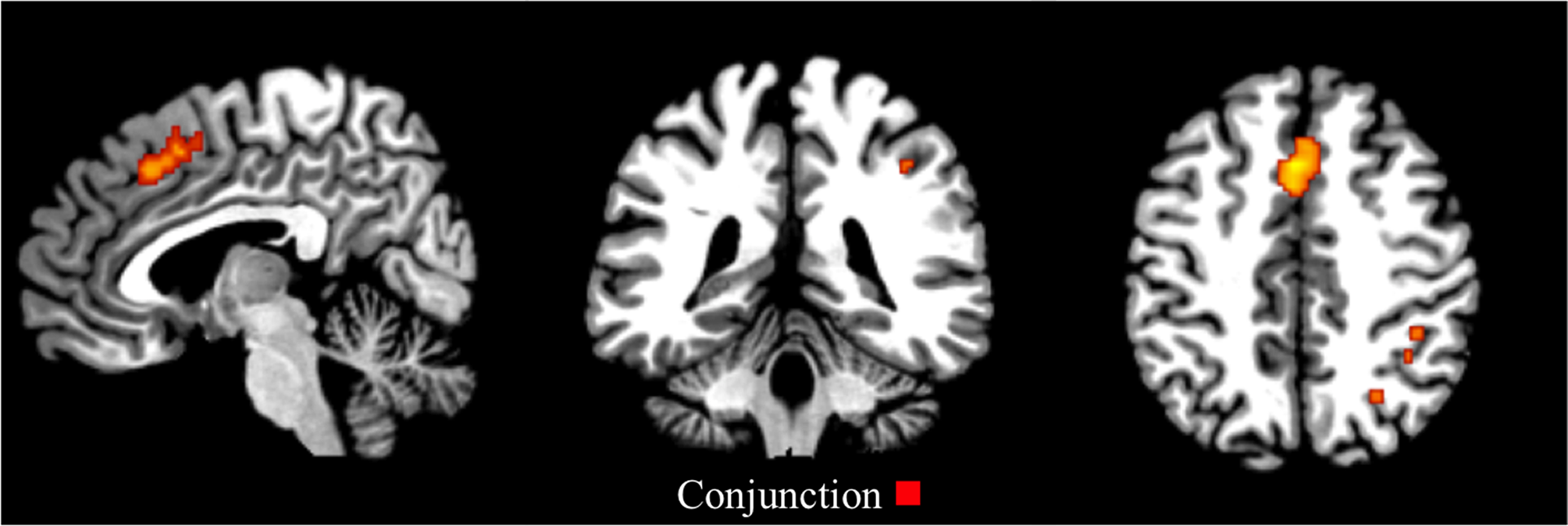

Supplement: Supplementary file 3 [file Image2.TIFF]

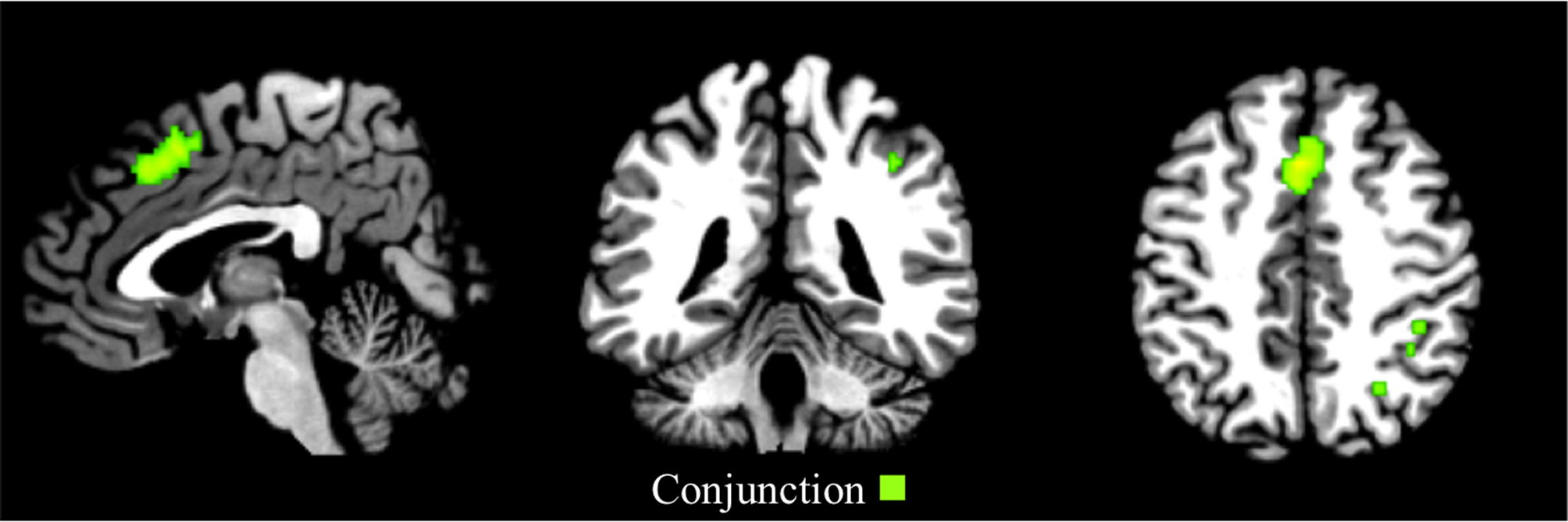

Supplement: Supplementary file 4 [file Image3.TIFF]

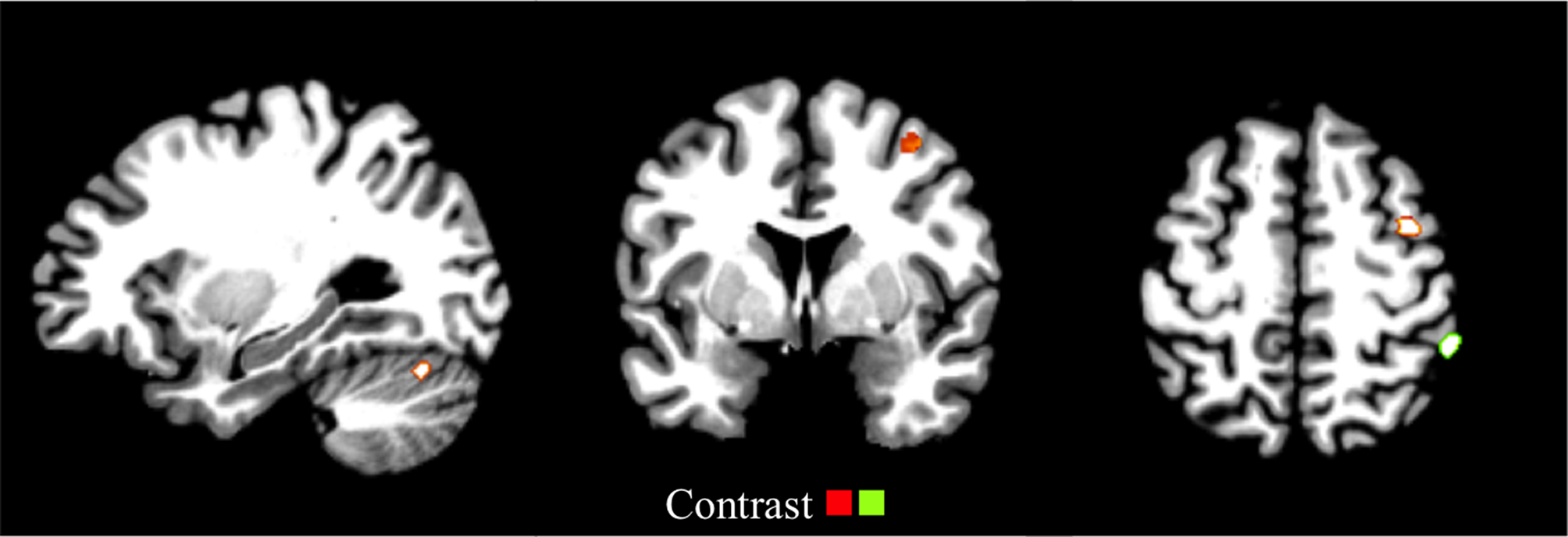

Supplement: Supplementary file 5 [file Image4.TIFF]
